# Prevalence, Characterization, and Proteomic Relatedness Among β-Lactam-Resistant Bacteria Throughout the Poultry Production Chain in Greece

**DOI:** 10.3390/foods14020224

**Published:** 2025-01-13

**Authors:** Anestis Tsitsos, Alexandros Damianos, Konstantinos Kiskinis, Vasilios Tsiouris, Ilias Tirodimos, Nikolaos Soultos, Anna Papa, Vangelis Economou

**Affiliations:** 1 Laboratory of Animal Food Products Hygiene and Veterinary Public Health, School of Veterinary Medicine, Aristotle University of Thessaloniki, 54124 Thessaloniki, Greece; tsitanes@vet.auth.gr (A.T.); adamianb@vet.auth.gr (A.D.); soultos@vet.auth.gr (N.S.); 2 Unit of Avian Medicine, Clinic of Farm Animals, School of Veterinary Medicine, Aristotle University of Thessaloniki, 54627 Thessaloniki, Greece; kiskinik@vet.auth.gr (K.K.); biltsiou@vet.auth.gr (V.T.); 3 Laboratory of Hygiene, Social & Preventive Medicine and Medical Statistics, School of Medicine, Aristotle University of Thessaloniki, 54124 Thessaloniki, Greece; ityrodim@auth.gr; 4 Laboratory of Microbiology, School of Medicine, Aristotle University of Thessaloniki, 54124 Thessaloniki, Greece; annap@auth.gr

**Keywords:** antibiotic resistance, ESKAPE, β-lactamase, multidrug resistance, poultry production chain, environment, poultry farmers, One Health

## Abstract

Extended-spectrum-β-lactamase (ESBL) and carbapenemase-producing *Escherichia coli*, *Klebsiella pneumoniae* and *Acinetobacter* spp. are associated with hospital-acquired infections and are commonly isolated across the poultry food production chain. Comprehensive data regarding the prevalence, spatiotemporal variations, and characterization of β-lactam-resistant bacteria in poultry farms and slaughterhouses is scarce. This study examines the prevalence and characteristics of β-lactam-resistant *E. coli*, *K. pneumoniae*, and *Acinetobacter* spp. isolated from poultry farms, slaughterhouses, and associated personnel in Greece. Strains were selectively isolated and identified via MALDI-TOF MS, which was also employed to identify possible relatedness. *E. coli* isolates were further classified into phylogenetic groups. The prevalence of β-lactam-resistant strains in farm and slaughterhouse environments was 15.0% (n = 15 strains)/57.3% (n = 71 strains) for *E. coli*, 11.0% (n = 11 strains)/1.6% (n = 2 strains) for *K. pneumoniae*, and 1.0% (n = 1 strain)/25.8% (n = 38 strains) for *Acinetobacter* spp., respectively. The prevalence of *Acinetobacter* spp. and *E. coli* on farmers’ skin was 16.7% (n = 2 strains) and 8.3% (n = 1 strain), correspondingly. Significantly higher *E. coli* isolation rates were observed in warmer seasons. All strains were multidrug-resistant and most carried ESBL/AmpC genes. Most *E. coli* isolates belonged to phylogroups A (41.4%, n = 36) and B1 (24.1%, n = 21). Proteomic analysis indicated relatedness among strains from different regions and seasons. Thus, poultry farms and slaughterhouses may serve as significant reservoirs of β-lactam-resistant strains of *E. coli*, *K. pneumoniae*, and *Acinetobacter* spp.

## 1. Introduction

Antimicrobial resistance (AMR) stands as one of the foremost global health crises of the 21st century, contributing to heightened rates of morbidity, mortality, and escalating healthcare expenditures [1]. The increasing rise of AMR significantly narrows the therapeutic choices against a broadening spectrum of bacterial infections, rendering once-effective treatments ineffective [2]. AMR, a natural evolutionary process fueled by genetic alterations in microorganisms, is provoked by the improper or excessive use of antimicrobials in both human and veterinary medicine [3]. Among the various types of AMR, resistance to β-lactams warrants particular scrutiny. Such resistance arises from various mechanisms including reduced membrane permeability, heightened efflux pump activity, target site modifications, and enzymatic inactivation by β-lactamases [4]. The production of β-lactamases is of interest, since the production of these enzymes is encoded by genes harbored on mobile genetic elements that can be transferred horizontally among both pathogenic and commensal microorganisms [5]. The most important β-lactamases are extended-spectrum β-lactamases (ESBLs), AmpC cephalosporinases, and carbapenemases. ESBL and AmpC-producing bacteria exhibit resistance not only to β-lactam antibiotics, but frequently to additional antibiotic classes [6]. Bacteria that produce carbapenemases are resistant to carbapenems, which are considered as last-resort antibiotics, and thus, the available treatment options are limited [7]. The production of β-lactamases is mainly exhibited by Gram-negative bacteria, such as species of the order Enterobacterales and *Acinetobacter baumannii*.

The order Enterobacterales encompasses a diverse group of bacterial species, ubiquitously present in environmental, animal, and human gut ecosystems. While often commensal, many of these species possess pathogenic potential, capable of causing infections in both healthcare and community settings [8]. Notably, *Escherichia coli* and *Klebsiella pneumoniae*, prominent members of the Enterobacterales order, frequently develop resistance to β-lactam antibiotics. ESBL and AmpC-producing strains of *E. coli* and *K. pneumoniae* are implicated in common infections, like urinary tract infections, and they may originate from human and non-human sources, such as animal-derived foods, which can colonize and infect humans [9,10]. Managing infections caused by these resistant strains poses challenges, given the limited antibiotic options available, often necessitating the use of carbapenems [11]. However, resistance to carbapenems is also escalating. Notably, KPC-producing *K. pneumoniae* is a prevalent carbapenemase-producing pathogen that can produce enzymes that degrade carbapenems [12]. Similarly, *Acinetobacter* spp. within the *A. baumannii–calcoaceticus* complex (ACB complex) frequently exhibit carbapenem resistance attributed to acquired carbapenemase production, notably OXA-type enzymes [13]. Strains within the ACB complex, particularly *A. baumannii*, are implicated in infections affecting various sites, including the bloodstream, urinary tract, lower respiratory tract (pneumonia), and wounds [14]. Despite their prevalence in environmental niches and largely saprophytic nature, environmental strains can colonize human body sites, precipitating hospital-acquired infections, particularly in immunocompromised individuals [15].

The environment acts as a primary reservoir for antibiotic-resistant bacteria [16]. Resistant strains of *E. coli*, *K. pneumoniae*, and *A. baumannii* to β-lactams have been identified in natural ecosystems (e.g., soil, water, sewage) and in human settings (e.g., hospitals, food industries) [17]. Natural ecosystems are considered genetic reservoirs, characterized by a rich diversity that exceeds that of both human and animal microbiomes [18]; within these ecosystems, microbial communities harbor a multitude of β-lactamase genes. Moreover, environmental bacteria frequently act as vectors for the dissemination of novel resistance genes among susceptible bacterial populations, a phenomenon more prevalent to certain natural and human environments, compared to their human and animal counterparts [19]. Within the spectrum of food production industries and animal-derived food products, poultry and its derivatives stand out as prominent reservoirs of β-lactamase-producing microorganisms. Poultry farming is characterized as the most intensive facet of animal husbandry, exposing poultry to elevated vulnerability to infections and compromised immune responses. The high population concentrations inherent in poultry farming facilitate the rapid proliferation of bacteria among flocks and their surrounding environment [1,20].

Despite the pivotal role of the poultry environment in disseminating β-lactam-resistant bacteria and β-lactamase genes, there is scarce information about the prevalence of ESBL, AmpC, and carbapenemase-producing Gram-negative bacteria, such as *E. coli*, *K. pneumoniae*, and *Acinetobacter* spp. of the ACB complex, in poultry farms and slaughterhouses. Greece is one of the Mediterranean countries that has been associated with the emergence of numerous novel ESBL, AmpC, and carbapenemase genes [21]. The presence of β-lactam-resistant bacteria in poultry environments can lead to cross-contamination and subsequent contamination of poultry meat if proper hygiene measures are not applied, thereby exposing humans to these bacteria and posing a public health risk. The present study evaluated the prevalence and seasonal fluctuations of β-lactamase-producing *E. coli*, *K. pneumoniae*, and *Acinetobacter* spp. of the ACB complex across the poultry food production chain in Greece, encompassing poultry farms, slaughterhouses, and the individuals working therein, based on the principle of One Health. This involves selective isolation with β-lactam antibiotics, subsequent characterization of the isolated strains, and investigation of potential malpractices that influence the prevalence of resistant strains from both human and environmental sources.

## 2. Materials and Methods

### 2.1. Sampling

**Figure 1:**
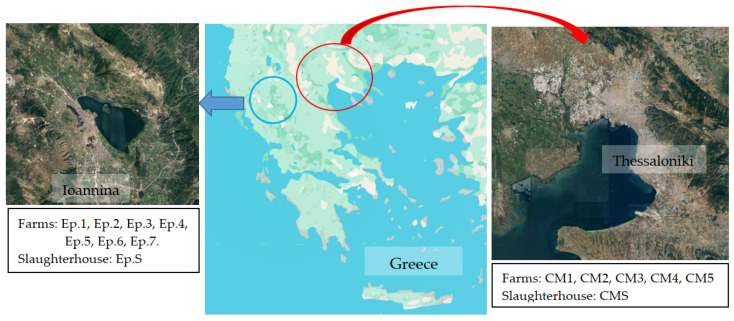
Map of Greece, showing the locations of poultry farms and slaughterhouses sampled in this study.

The study was conducted on several poultry farms and two slaughterhouses situated in Central Macedonia and Epirus (Figure 1). These regions in Greece were selected due to their notable concentration of poultry farms and high poultry meat production. The selected poultry farms are situated within a 40 km radius of designated study slaughterhouses, where animals from these farms are dispatched. Slaughterhouses were selected based on their annual poultry slaughter volume. The weekly capacity of the slaughterhouses in Epirus and Central Macedonia is approximately 500,000 and 100,000 poultry, respectively. Sampling was performed at the end of each season between winter 2023 and autumn 2023. Each season, environmental samples were collected from five poultry farms in each region and the corresponding slaughterhouses (Table S1).

In total, 224 environmental samples were gathered, with 100 samples originating from poultry farm settings and 124 from slaughterhouse environments. At poultry farms, samples were collected from watering troughs, cooling pads, ventilation fans, and poultry litter. Meanwhile, slaughterhouse samples included chicken transport boxes, pluckers, evisceration machines, carcass cutting equipment, cutting boards, drains, and sewage (influent and effluent). Sampling at poultry farms occurred after the initial thinning of the flock, a period associated with heightened stress in birds and thus, a potentially increased excretion of resistant microorganisms into the environment. Sampling within slaughterhouses took place during the slaughtering and carcass preparation processes of animals from designated study poultry farms. The sampling was evenly distributed across poultry farms, slaughterhouses, and seasons (Table 1). Sample collection was conducted using a pair of sterile gauzes or swabs soaked in 5 mL Buffered Peptone Water (BPW, Oxoid, Basingstoke, UK) to wipe 100 cm^2^ surface areas. Poultry litter samples were collected using boot swabs, while sewage sampling involved collecting 500 mL of influent sewage and 4 L of effluent sewage in sterile bottles with screw caps containing 18 mg/L or 54 mg/L sodium thiosulfate, respectively. All samples were promptly transported in a portable refrigerator (<4 °C) to the laboratory for further processing within 24 h.

Additionally, 80 samples were obtained from breeders and workers at the slaughterhouse who have direct contact with poultry flocks or their carcasses. Specifically, samples were obtained from the skin (hands and arms) and oropharyngeal regions of 12 poultry breeders (5 in Central Macedonia and 7 in Epirus) and 20 abattoir workers (10 from each region). For skin sampling, a pair of sterile swabs, one dry and one moistened in 5 mL BPW, were used to swab both hands thoroughly. Oropharyngeal samples were collected using a sterile swab. Then, the swabs were placed in tubes with 5 mL BPW. In 8 poultry breeders (5 in Central Macedonia and 3 in Epirus), human sample collection was conducted biannually, with a repeat sampling six months after the initial collection. Before sampling, poultry breeders responded to a series of questions regarding the medical history and the history of antibiotic consumption for themselves and the poultry flock (Tables S2 and S3) to identify potential behaviors and factors contributing to resistant strain occurrence in humans and the environment, based on the principle of One Health.

### 2.2. Microbiological Examination, Identification, and Proteomic Relationship of the Isolated Strains

The experimental procedure aimed to isolate resistant bacteria and specifically strains producing ESBL, AmpC, and carbapenemase. The methods followed were outlined by EFSA [22,23] and Carvalheira et al. [24], with some adjustments. Initially, boot swabs used for sampling poultry litter were placed into stomacher bags (Interscience, Saint Nom la Bretêche, France) containing sterile BPW and homogenized for 2 min in a stomacher (Interscience, Saint Nom la Bretêche, France). Afterwards, both the stomacher bags and test tubes containing environmental and human swabs were incubated at 37 °C for 2 h, serving as a pre-enrichment step for microorganism recovery. Subsequently, 100 μL of the enriched rinsates were transferred into 10 mL McConkey or Dijkshoorn broth (Oxoid, Basingstoke, UK), supplemented with ceftazidime (1 mg/L) or meropenem (0.125 mg/L). McConkey broth with ceftazidime or meropenem was utilized for isolating resistant *E. coli*, *K. pneumoniae*, and *Acinetobacter* spp., while Dijkshoorn broth, supplemented with antibiotics, was employed as an additional selective enrichment medium for isolating resistant *Acinetobacter* spp. [24]. Regarding sewage analysis, 1 mL of influent sewage was directly transferred into 100 mL of McConkey or Dijkshoorn broth supplemented with antibiotics. The analysis of effluent sewage involved filtering 1 L of sewage through sterile microbiological membranes with a pore size of 0.45 μm (CHMLAB GROUP, Barcelona, Spain). The membranes were then transferred into sterile tubes with 50 mL McConkey or Dijkshoorn broth, supplemented with antibiotics. McConkey broth was incubated at 37 °C for 24 h, while Dijkshoorn broth was incubated at 30 °C for 24–48 h. Following enrichment, 10 μL of McConkey cultures were plated onto McConkey agar supplemented with 0.125 mg/L meropenem or 1 mg/L ceftazidime and Chromocult TBX agar (Merck GmbH, Darmstadt, Germany), and were then incubated at 37 °C and 44 °C, respectively, for 24 h. Similarly, 10 μL of Dijkshoorn broth was surface-inoculated onto CHROMagarTM Acinetobacter supplemented with 0.125 mg/L meropenem or 1 mg/L ceftazidime, followed by incubation at 30 °C for 24–48 h. From each plate, up to five characteristic colonies were subcultured on Tryptic Soy Agar (TSA, Oxoid, Basingstoke, UK).

The isolated strains were identified through matrix-assisted laser desorption/ionization-time-of-flight mass spectrometry (MALDI-TOF MS) using a Microflex LT spectrometer (Bruker Daltonics, Bremen, Germany). Protein extraction from selected *E. coli*, *K. pneumoniae*, and *Acinetobacter* spp. colonies was performed using the formic acid method according to the manufacturer’s instructions. Single colonies were homogenized in 300 μL of ultrapure water, mixed with 900 μL of pure ethanol, centrifuged at 13,000 rpm for 2 min, and subjected to repeated ethanol washes. After ethanol evaporation, protein extracts were treated with 30 μL of 70% formic acid and 30 μL of acetonitrile. After centrifugation at 13,000 rpm for 2 min, 1 μL of the protein extract was applied onto the MALDI-TOF MS target plate. Finally, 1 μL of a saturated solution of cyano-4-hydroxycinnamic acid matrix (Bruker Daltonics) in 50% acetonitrile (SigmaAldrich, St. Louis, MO, USA) and 25% trifluoroacetic acid (SigmaAldrich) was overlaid onto each sample and air-dried.

Protein profiles were obtained in linear positive mode with a 20 Hz laser frequency over a mass range of 2000–20,000 Da using AutoXecute software (Flex Control 3.4; Bruker Daltonics). The spectra were calibrated against the Bruker Bacterial Test Standard (BTS), which is a protein extract from *E. coli* DH5 bacteria that is supplemented with two additional proteins (RNAase A and myoglobin), in order to expand the mass range covered by BTS. Then, the spectra were analyzed using MALDI Biotyper software version 4.0, which compared them to the reference library (v6.093 MSPs). The adjusted score values were used for the categorization of the results, following the manufacturer’s guidelines. Each spectrum underwent baseline subtraction and smoothing using the MALDI Biotyper Offline Classification 4.0 software with default parameters. A dendrogram illustrating Main Spectra Profiles (MSPs) was generated, clustering strains based on a cut-off distance of 400 for optimal differentiation as proposed by Tsitsos et al. [25]. Isolated strains of *E. coli*, *K. pneumoniae*, and *Acinetobacter* spp. of the ACB complex were preserved at −80 °C with 15% glycerol for further analysis.

### 2.3. Antimicrobial Susceptibility Testing of the Isolated Strains

The antibiotic susceptibility of the isolated strains was assessed using the disc diffusion method, following the guidelines set by the Clinical and Laboratory Standards Institute [26]. From an overnight culture on TSA, two or three pure colonies were selected and suspended in 10 mL of sterile normal saline. The turbidity of the suspension was adjusted to 0.5 McFarland scale using a nephelometer (Densitomat, bioMérieux, France), approximately equal to 10^8^ CFU/mL. Next, an aliquot was streaked onto Mueller Hinton agar plates using a sterile swab (bioMérieux, Marcy-l’Étoile, France), followed by incubation at 35 °C for 24 h. The susceptibility of *E. coli* and *K. pneumoniae* strains was assessed against 22 antibiotics, whereas the susceptibility of *Acinetobacter* strains was evaluated against 16 antibiotics, commonly used in medical and veterinary practice (Oxoid, Basingstoke, UK) (Table S4). The quality control strain *E. coli* ATCC 25922 validated the testing process. The diameter of inhibition zones was measured to the nearest millimeter (mm) using a caliper. Following criteria established by Magiorakos et al. [27], strains displaying resistance to at least one antimicrobial agent from three or more categories were categorized as multidrug-resistant (MDR). Additionally, strains resistant to at least one antimicrobial agent from all but ≤2 categories were classified as extensively drug-resistant (XDR), while those exhibiting resistance to all antimicrobial agents were considered pandrug-resistant (PDR).

Additionally, colistin susceptibility was evaluated using a broth microdilution method in accordance with CLSI guidelines [26]. Initially, stock solutions of colistin sulfate (Sigma-Aldrich, Saint Louis, MO, USA, 1280 μg in 1 mL of sterile water) were prepared, and concentrations from 0.06 to 32 μg/mL were utilized to determine the Minimum Inhibitory Concentration (MIC) of colistin. The 0.5 McFarland inoculum suspensions were diluted further by a factor of 1:100 in Mueller–Hinton broth before inoculation. Each well of the 96-well microtiter test plates was filled with 50 μL of Mueller–Hinton broth containing a twofold concentration of colistin, followed by the addition of 50 μL of the suspended culture to achieve a final inoculum density of 5 × 10^5^ CFU /mL per well. Positive controls (bacterial culture without colistin) and negative controls (culture medium only) were incorporated for each strain on the plates, and each isolate was tested in triplicate. The plates were sealed with parafilm and incubated at 35 °C for 24 h. MICs of colistin were determined as the lowest concentration at which no visible growth was observed in the wells of the microtiter plates. *E. coli* ATCC 25922 served as the quality control strain.

### 2.4. Confirmation of β-Lactamase Production

The phenotypic characterization of the *E. coli* and *K. pneumoniae* isolates was performed with the combination disk test, based on CLSI [26] and EUCAST [28] protocols. Inocula were prepared and spread on Mueller–Hinton agar plates (bioMérieux, France), followed by the application of antibiotic disks (Himedia, Mumbai, India) containing cefotaxime (CTX 30 μg), cefotaxime/clavulanic acid (CTC 30/10 μg), ceftazidime (CAZ 30 μg), ceftazidime/clavulanic acid (CZC 30/10 μg), and cefoxitin (FOX 30 μg). ESBL phenotype was confirmed by a ≥5 mm increase in inhibition zone diameter when clavulanic acid was combined with ceftazidime or cefotaxime, compared to the antibiotic alone. AmpC phenotype was indicated by cefoxitin resistance and resistance to ceftazidime and cefotaxime without clavulanic acid induction. Combined ESBL and AmpC phenotypes were identified by cefoxitin resistance, along with an increase of ≥5 mm in the zone diameter when combining the antibiotic with clavulanic acid. *K. pneumoniae* ATCC 700603 and *E. coli* ATCC 25922 served as quality control strains.

### 2.5. Molecular Screening of β-Lactamase Genes

All β-lactam-resistant *Acinetobacter* strains, as well as *E. coli* and *K. pneumoniae* isolates, were confirmed to display an ESBL or AmpC phenotype, underwent additional molecular analysis for β-lactamase gene identification. DNA extraction followed the methodology of Peratikos et al. [29], involving heat lysis (100 °C for 10 min) of pure colonies in 50 μL of sterile Milli-Q water, cooling on ice, and centrifugation for 5 min at 13,000 rpm. The supernatant was collected and stored at −20 °C in a new tube. A NanoDrop microvolume spectrophotometer (Nanodrop 2000, Thermo Fisher Scientific, Waltham, MA, USA) was used for the evaluation of DNA quality and yield. Subsequently, four multiplex PCRs and one simplex PCR were employed to screen for common ESBL and AmpC genes, following the protocol described by Dallenne et al. [30] (Table S5). The reaction mixture, prepared to a final volume of 25 μL, consisted of 0.2–0.5 μM of primers, 200 μM dNTPs (N0447S, NEB, Ipswich, MA, USA), 0.625U of OneTaq™ DNA Polymerase (M0273S, NEB), 2.5 μL of 10× OneTaq Standard Reaction Buffer (B9014S, NEB), and 2 μL of DNA sample (Table S5). PCR was carried out in a thermal cycler (LabCycler Gradient, SensoQuest GmbH, Göttingen, Germany) with an initial denaturation at 95 °C for 3 min, followed by 30 cycles of denaturation at 95 °C for 30 s, annealing at 60 °C for 40 s (54 °C for the simplex PCR), and extension at 68 °C for 1 min. A final extension step was conducted at 68 °C for 5 min. *K. pneumoniae* ATCC 700603 was utilized as the positive control. After electrophoresis on 1.5% agarose gels, the PCR products were visualized using a UVP DigiDoc-It^®^ 125 gel imaging system (UVP, Cambridge, UK) after staining with ethidium bromide.

### 2.6. Phylogenetic Analysis of E. coli Isolates

The PCR protocols developed by Clermont et al. [31] were utilized with modifications to determine the phylogenetic groups of the *E. coli* isolates (Tables S6 and S7). Each reaction mixture, totaling 25 μL, consisted of 0.2–0.4 μM of primers, 200 μM dNTPs (N0447S, NEB), 0.625U OneTaq™ DNA Polymerase (M0273S, NEB), 2.5 μL 10× OneTaq Standard Reaction Buffer (B9014S, NEB), and 2 μL of DNA sample (Table S6). The PCR process commenced with an initial denaturation at 95 °C for 180 s, followed by 30 PCR cycles comprising denaturation at 95 °C for 30 s, annealing at either 59 °C for 20 s (for quadruplex PCR and PCR of group C) or 57 °C for 20 s (for PCR of group E), and a final extension step at 68 °C for 60 s. A final extension was performed at 68 °C for 5 min. *E. coli* ATCC 25922 served as the positive control. Subsequently, the PCR products were observed via electrophoresis on 1.5% agarose gels containing ethidium bromide using a UVP DigiDoc-It^®^ 125 gel imaging system (UVP, Cambridge, UK).

### 2.7. Statistical Analysis

Data were analyzed using IBM SPSS Statistics software (v.29.0., IBM Corporation, Armonk, NY, USA). Summary statistics, including measures of central tendency and dispersion, were calculated to characterize the data. The Clopper–Pearson method (Binomial test) provided confidence intervals for prevalence estimates. Comparisons of the prevalence of resistant strains in environmental and human samples across seasons were conducted using Fisher’s exact test. Fisher’s exact test was also used to compare questionnaire variables between breeders or poultry farms that tested positive for resistant strains and those that tested negative. A significance level of 5% (*p*-value ≤ 0.05) was applied.

## 3. Results

### 3.1. Prevalence, Seasonal, and Regional Variations in Isolates

**Figure 2:**
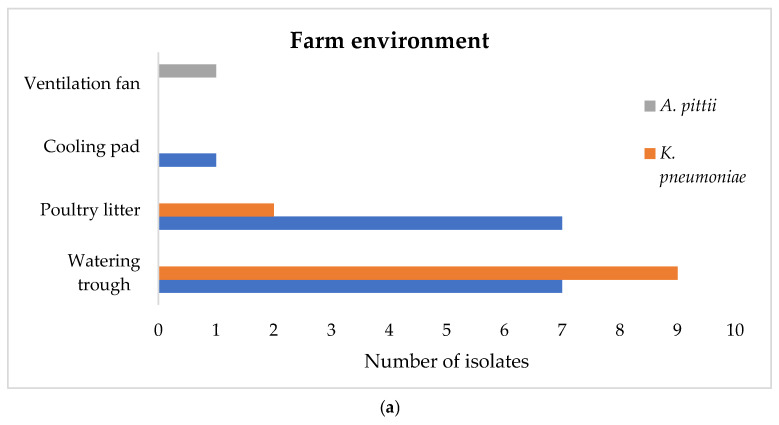

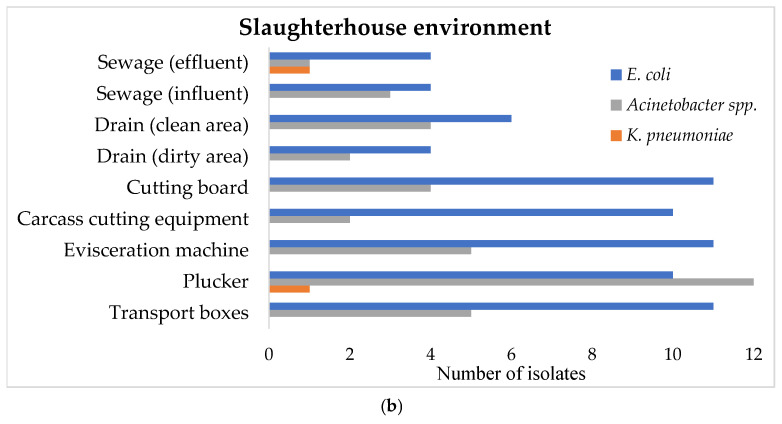
Number of strains isolated across the sampling points in the farm (**a**) and slaughterhouse environment (**b**).

In the farm environment, among 100 samples tested, 25 were found positive for ceftazidime-resistant strains of *E. coli*, *K. pneumoniae*, or *Acinetobacter* spp., with no detection of meropenem-resistant isolates. Specifically, 15 samples harbored ceftazidime-resistant *E. coli* (15.0%, 95% CI = 8.6–23.5%), 11 contained ceftazidime-resistant *K. pneumoniae* (11.0%, 95% CI = 5.6–18.8%), and 1 sample carried ceftazidime-resistant *Acinetobacter* spp. (1.0%, 95% CI = 0.0–5.4%). Two samples contained both resistant *E. coli* and *K. pneumoniae* isolates. Each sample yielded one strain per bacterial species, totaling 27 isolates from the farm environment: 15 *E. coli* (55.6%), 11 *K. pneumoniae* (40.7%), and 1 *A. pittii* (3.7%) isolate (Figure 2, Tables S8–S10). Concerning the slaughterhouse environment, out of 124 samples tested, 88 were positive for ceftazidime-resistant strains of *E. coli*, *K. pneumoniae*, or *Acinetobacter* spp., whereas no meropenem-resistant strains were detected. Specifically, 71 samples showed presence of ceftazidime-resistant *E. coli* (57.3%, 95% CI = 48.1–66.1%), two contained ceftazidime-resistant *K. pneumoniae* (1.6%, 95% CI = 0.2–5.7%), and 32 samples carried ceftazidime-resistant *Acinetobacter* spp. (25.8%, 95% CI = 18.4–34.4%). Moreover, one sample exhibited co-presence of resistant *E. coli* and *K. pneumoniae*, while 22 samples contained both resistant *E. coli* and *Acinetobacter* spp. In total, 111 ceftazidime-resistant strains were isolated, comprising 71 *E. coli* isolates (64.0%), 2 *K. pneumoniae* isolates (1.8%), and 38 *Acinetobacter* spp. of the ACB complex (34.2%). Among the 38 *Acinetobacter* spp., 37 were identified as *A. baumannii* and 1 as *A. pittii*, isolated from a transport box (Figure 2, Tables S8–S10).

No significant differences were observed in the prevalence of resistant *K. pneumoniae* and *Acinetobacter* spp. in the farm environment (*p*-values = 0.11 and 1.00, respectively) across seasons. Specifically, one strain of *K. pneumoniae* was isolated in winter (4.0% of samples, 95% CI = 0.1–20.4%), three in spring (12.0%, 95% CI = 2.5–31.2%), one in summer (4.0%, 95% CI = 1.0–20.4%), and six in autumn (24.0%, 95% CI = 9.4–45.1%). For *Acinetobacter* spp., one isolate was recovered in spring (4.0%, 95% CI = 0.1–20.4%), and none were recovered in the other seasons (95% CI = 0.0–13.7%). In contrast to *K. pneumoniae* and *Acinetobacter* spp., seasonality significantly influenced the prevalence rates of *E. coli* in the farm environment (*p*-value = 0.003 < 0.05). Most *E. coli* strains were isolated in the summer (9 strains, 36.0% of samples, 95% CI = 18.0–57.5%), followed by winter (4 strains, 16.0%, 95% CI = 4.5–36.1%) and spring (2 strains, 8.0%, 95% CI = 1.0–26.0%), whereas none were isolated in autumn (95% CI = 0.0–13.7%). Similarly, the prevalence rates of *E. coli* in the farm environment were significantly higher in the Central Macedonia region (13 isolates, 26.0% of samples, 95% CI = 14.6–40.3%) compared to the Epirus region (2 isolates, 4.0%, 95% CI = 0.5–13.7%) (*p*-value = 0.004 < 0.05). However, no significant differences were observed in the prevalence of resistant *K. pneumoniae* and *Acinetobacter* spp. in the farm environment (*p*-values = 0.051 and 1.00, respectively) between the two regions. Two (4.0% of samples, 95% CI = 0.5–13.7%) and nine (18.0%, 95% CI = 8.6–31.4%) strains of *K. pneumoniae* were isolated in the Central Macedonia and Epirus regions, respectively, whereas one strain of *Acinetobacter* spp. was isolated in the Epirus region (2.0%, 95% CI = 0.1–10.6%) and none in the Central Macedonia region (95% CI = 0.0–7.1%).

Similarly to the farm environment, seasonality significantly influenced the prevalence rates of *E. coli* in the slaughterhouse environment (*p*-value = 0.03 < 0.05). However, no significant differences were observed in the prevalence of resistant *K. pneumoniae* and *Acinetobacter* spp. in the slaughterhouse environment across seasons (*p*-values = 0.24 and 0.41, respectively). Specifically, 11 strains of *E. coli* were isolated in winter (35.5% of samples, 95% CI = 19.2–54.6%), 22 in spring (71.0%, 95% CI = 52.0–85.8%), 20 in summer (64.5%, 95% CI = 45.4–80.8%), and 18 in autumn (58.1%, 95% CI = 39.1–75.5%). For *K. pneumoniae*, two strains were isolated in summer (6.5% of samples, 95% CI = 0.8–21.4%), whereas none were isolated in the other seasons (95% CI = 0.0–11.2%). Regarding *Acinetobacter* spp., 13 isolates were recovered from eight samples in winter (25.8% of samples, 95% CI = 11.9–44.6%), and six isolates from five samples in summer (16.1%, 95% CI = 5.5–33.7%). In spring, eight strains were isolated (25.8%, 95% CI = 11.9–44.6%), whereas in autumn 11 isolates were recovered (35.5%, 95% CI = 19.2–54.6%). Moreover, no significant differences were observed in the prevalence rates of *E. coli*, *K. pneumoniae*, and *Acinetobacter* spp. between the two regions (*p*-value = 0.15, 0.50, and 0.31, respectively). In the Central Macedonia region, the prevalence rates of resistant *E. coli* and *K. pneumoniae* isolates were 50.0% (31 isolates, 95% CI = 37.0–63.0%) and 3.2% (2 isolates, 95% CI = 0.4–11.2%), respectively, whereas 19 isolates of *Acinetobacter* spp. were recovered from 13 samples (21.0% of samples, 95% CI = 11.7–33.2%). In the Epirus region, the prevalence rates of resistant *E. coli* and *Acinetobacter* spp. isolates were 64.5% (40 isolates, 95% CI = 51.3–76.3%) and 30.6% (19 isolates, 95% CI = 19.6–43.7%), correspondingly, whereas no *K. pneumoniae* strains were isolated (95% CI = 0.0–5.8%).

In human samples, two *A. baumannii* strains were isolated from the skin of two poultry breeders (16.7% of breeders, 95% CI = 2.1–48.4%), and one *E. coli* strain was isolated from the skin of one poultry breeder (8.3%, 95% CI = 0.2–38.5%) (Tables S8–S10). Interestingly, the *E. coli* strain was concurrently isolated with one *A. baumannii* strain from the skin of a poultry breeder in the Central Macedonia region during the summer. The other *A. baumannii* isolate was recovered from a poultry breeder in the Epirus region during autumn. No *K. pneumoniae* isolates were retrieved from the skin of poultry breeders (95% CI = 0.0–26.5%). Moreover, no isolates were obtained from the oropharynx of poultry breeders (95% CI = 0.0–26.5%), as well as from the skin and oropharynx of workers in the slaughterhouse (95% CI = 0.0–16.8%). Still, no significant differences were noted in the prevalence rates of *E. coli* and *Acinetobacter* spp. between the skin of poultry breeders and workers in the slaughterhouse (*p*-value = 0.38 and 0.13, respectively). Furthermore, no significant differences were observed in the prevalence rates of *E. coli* and *Acinetobacter* spp. across seasons (*p*-value = 1.00) and regions (*p*-value = 0.42 and 1.00, respectively).

### 3.2. Antimicrobial Susceptibility Testing of the Isolated Strains

**Figure 3:**
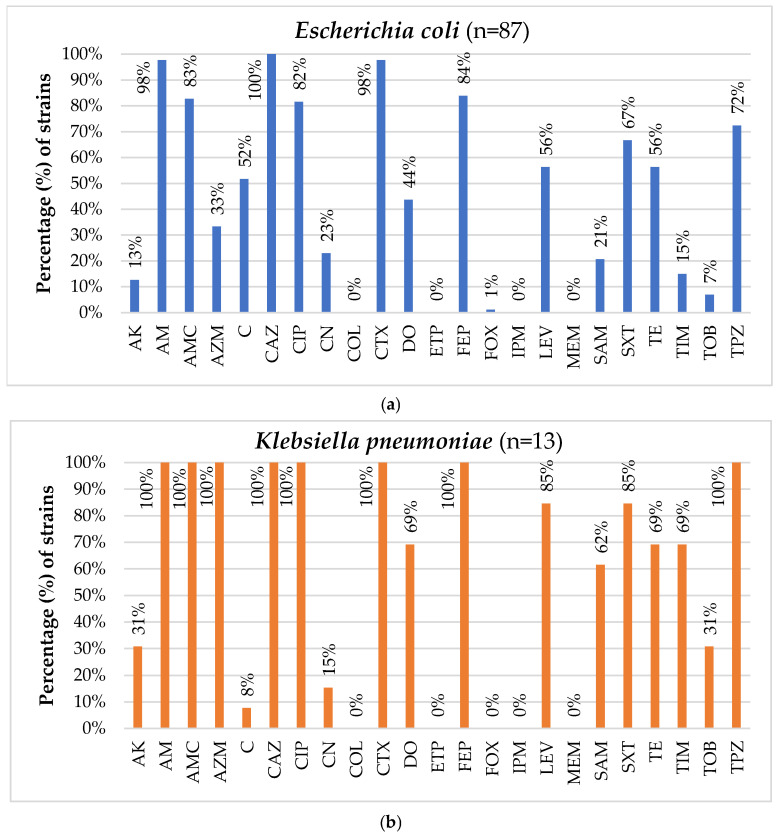

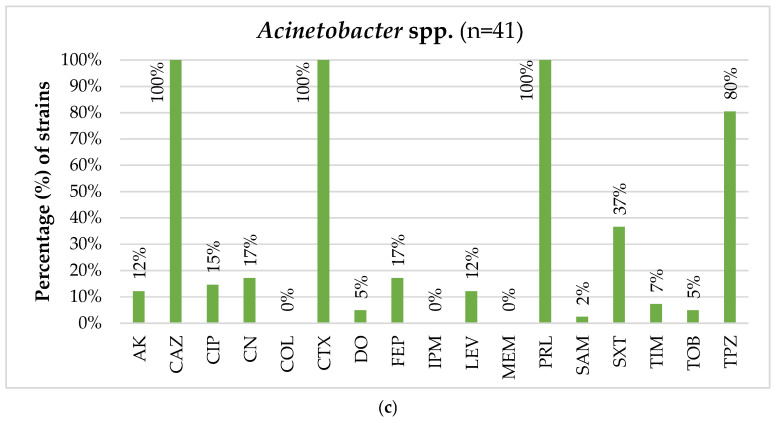
AMR profiles of the (**a**) *E. coli* (n = 87), (**b**) *K. pneumoniae* (n = 13), and (**c**) *Acinetobacter* (n = 41) isolates.

Regarding the *E. coli* isolates, the spectrum of antibiotic resistance ranged from 0.0% (against MEM, ETP, IPM, and COL) to 100.0% (against CAZ). Particularly noteworthy were the high resistance rates observed for AM (97.7%, n = 85), CTX (97.7%, n = 85), FEP (83.9%, n = 73), AMC (82.8%, n = 72), and CIP (81.6%, n = 71). The majority of the strains (85.7%, n = 42) exhibited MICs ≤ 0.06 μg/mL for colistin, while 15 and 2 strains demonstrated MICs of 0.125 μg/mL and 0.25 μg/mL for colistin, respectively. The strains exhibited resistance to different numbers of antibiotics, from 4 (2.1%, n = 2) to 16 distinct antibiotics (1.1%, n = 1). Consequently, all strains (100.0%) were classified as multidrug-resistant (MDR), showcasing resistance to at least three different antibiotic categories. In total, 71 distinct antimicrobial resistance profiles were identified. The most shared resistance profile was [AM, AMC, C, CAZ, CIP, CTX, FEP, LEV, SXT, TE, TPZ], observed in six strains (6.9%). Furthermore, the resistance profiles [AM, AMC, C, CAZ, CIP, CTX, FEP, LEV, SXT], [AM, AMC, C, CAZ, CIP, CTX, FEP, LEV, SXT, TE], and [AM, CAZ, CIP, CTX, DO, FEP, SXT, TE] were each shared by three isolates (3.4%), while the resistance profiles (a) [AK, AM, AMC, CAZ, CIP, CN, CTX, DO, FEP, SXT, TE, TPZ], (b) [AM, AMC, AZM, C, CAZ, CTX, DO, FEP, SXT, TE, TPZ], (c) [AM, AMC, C, CAZ, CIP, CTX, DO, FEP, LEV, SXT, TE, TPZ], (d) [AM, AMC, C, CAZ, CIP, CTX, FEP, LEV, SXT, TPZ], and (e) [AM, AMC, CAZ, CTX, FEP] were each shared by two isolates (2.3%) (Figure 3, Table S8).

Concerning *K. pneumoniae*, all isolates (100.0%) exhibited resistance to AM, AMC, AZM, CAZ, CIP, CTX, FEP, and TPZ, while high resistance rates were also observed for LEV (84.6%, n = 11), SXT (84.6%, n = 11), DO (69.2%, n = 9), TE (69.2%, n = 9), TIM (69.2%, n = 9), and SAM (61.5%, n = 8). Notably, all isolates were susceptible to FOX, MEM, IPM, ETP, and COL. The majority of *K. pneumoniae* strains (61.5%, n = 8) exhibited MICs ≤ 0.06 μg/mL for colistin, while five strains (38.5%) had an MIC value of 0.125 μg/mL. Like *E. coli*, all *K. pneumoniae* isolates were categorized as MDR, displaying resistance to 9–17 different antibiotics. Eleven distinct antimicrobial resistance profiles were identified, with the profiles [AK, AM, AMC, AZM, CAZ, CIP, CTX, DO, FEP, LEV, SAM, SXT, TE, TIM, TOB, TPZ] and [AM, AMC, AZM, CAZ, CIP, CTX, DO, FEP, LEV, SAM, SXT, TE, TIM, TPZ] being shared by two isolates each (15.4%) (Figure 3, Table S9).

All *Acinetobacter* spp. isolates (100.0%) exhibited resistance to CAZ, CTX, and PRL, while they also displayed high resistance rates to TPZ (80.5%, n = 33). Conversely, all strains demonstrated sensitivity to COL, IPM, and MEM. The median MIC for colistin was 0.125 μg/mL (IQR = 0.125). These isolates demonstrated varying degrees of resistance, with the lowest showing resistance to 3 antibiotics (12.2%, n = 5) and the highest to 12 antibiotics (4.9%, n = 2). Consequently, the majority of *Acinetobacter* isolates were classified as MDR (87.8%, n = 36). A total of 12 distinct antimicrobial resistance profiles were identified. The most prevalent profile was [CAZ, CTX, PRL, TPZ], shared by a little less than half of the isolates (43.9%, n = 18). Other common resistance profiles included [CAZ, CTX, PRL, SXT, TPZ] and [CAZ, CTX, PRL], shared by 7 (17.1%) and 5 isolates (12.2%), respectively, as well as [AK, CAZ, CIP, CN, CTX, FEP, LEV, PRL, SXT, TIM, TOB, TPZ] and [AK, CAZ, CN, CTX, FEP, PRL, SXT, TPZ], each shared by two isolates (4.9%) (Figure 3, Table S10).

### 3.3. Phenotype of β-Lactam Resistance and Molecular Screening of β-Lactamase Genes

**Figure 4:**
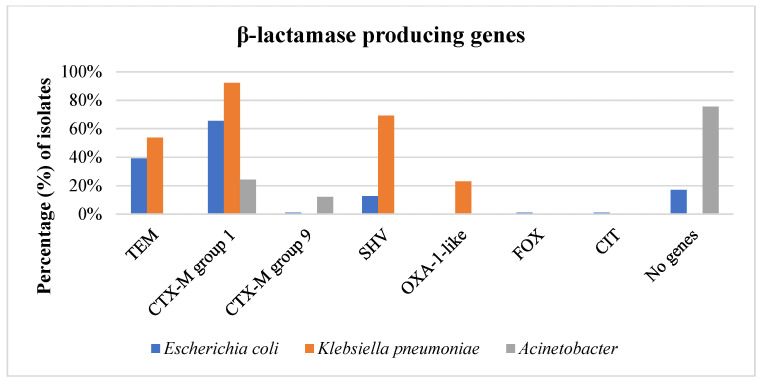
Detection of β-lactamase genes in *E. coli* (n = 87), *K. pneumoniae* (n = 13), and *Acinetobacter* (n = 41) isolates.

All identified *E. coli* and *K. pneumoniae* isolates resistant to β-lactams were phenotypically confirmed as β-lactamase producers. Among the *E. coli* isolates, 86 (98.8%) were confirmed as ESBL producers, while one strain (1.1%) was confirmed as both an ESBL and AmpC producer. Within the ESBL-producing *E. coli*, 28 harbored *bla*_CTX-M_ genes of group 1 (32.2%), 28 harbored both *bla*_TEM_ and *bla*_CTX-M_ genes of group 1 (32.2%), 9 harbored *bla*_SHV_ genes alone (10.4%), 3 carried *bla*_TEM_ genes alone (3.4%), and 2 carried both *bla*_TEM_ and *bla*_SHV_ genes (2.3%). Additionally, two ESBL-producing *E. coli* isolates (2.3%) were found to harbor *bla*_AmpC_ genes, despite not being phenotypically confirmed as AmpC producers. More specifically, one of these strains harbored *bla*_FOX_ and *bla*_CTX-M_ genes of group 1, while the other carried *bla*_TEM_, *bla*_CIT_, and *bla*_CTX-M_ genes of group 9. In 15 *E. coli* isolates (17.2%), including the ESBL + AmpC producer, no β-lactamase genes were detected (Figure 4, Table S8).

Regarding *K. pneumoniae*, all isolated strains (100.0%) were confirmed as ESBL producers. Approximately half of the strains harbored *bla*_TEM_, *bla*_SHV_ and *bla*_CTX-M_ genes of group 1 (6 strains, 46.1%), two strains carried *bla*_OXA-1_ and *bla*_CTX-M_ genes of group 1 (15.4%), and two strains harbored *bla*_SHV_ and *bla*_CTX-M_ genes of group 1 (15.4%). Moreover, one strain carried *bla*_TEM,_ *bla*_OXA-1_ and *bla*_CTX-M_ genes of group 1 (7.7%), one strain harbored *bla*_SHV_ alone (7.7%), and another strain harbored *bla*_CTX-M_ genes of group 1 alone (7.7%) (Figure 4, Table S9).

Among the 41 ceftazidime-resistant *Acinetobacter* strains, only 10 (24.4%) were found to carry a β-lactamase gene. Specifically, half of these strains (12.2%) harbored *bla*_CTX-M_ genes of group 1, and the rest carried *bla*_CTX-M_ genes of groups 1 and 9 (12.2%). Notably, no *bla*_TEM,_ *bla*_SHV_, *bla*_OXA_, or *bla*_AmpC_ genes were detected in the resistant *Acinetobacter* spp. (Figure 4, Table S10).

### 3.4. Proteomic Relationship of Isolates

**Figure 5:**
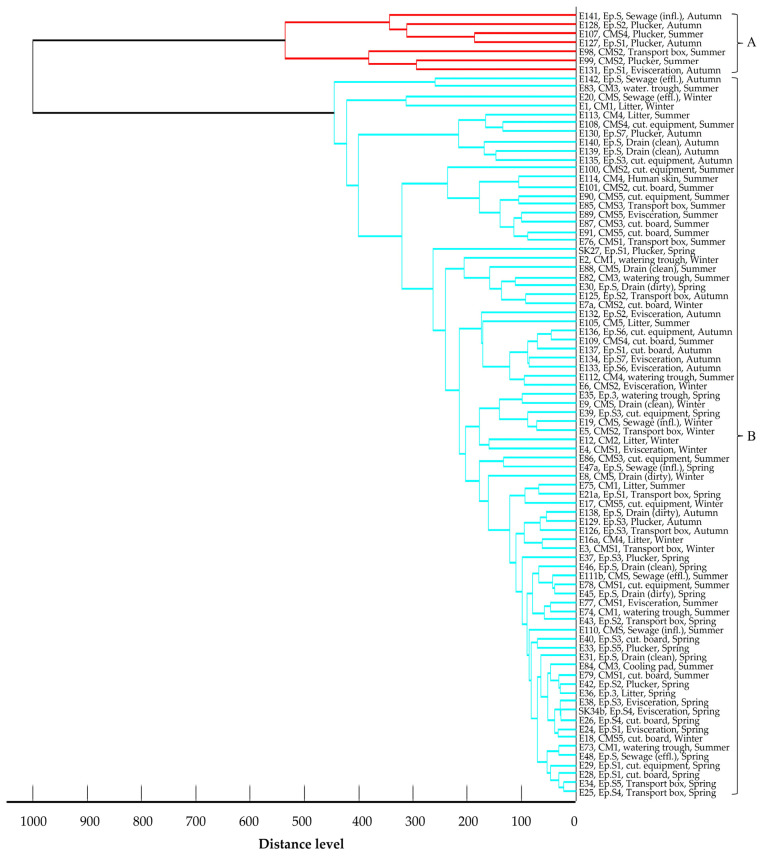
Main spectra dendrogram of *E. coli* isolates (strain I.D., origin, and season of isolation). The clusters are depicted by different colors in dendrogram branches and capital letters.

**Figure 6:**
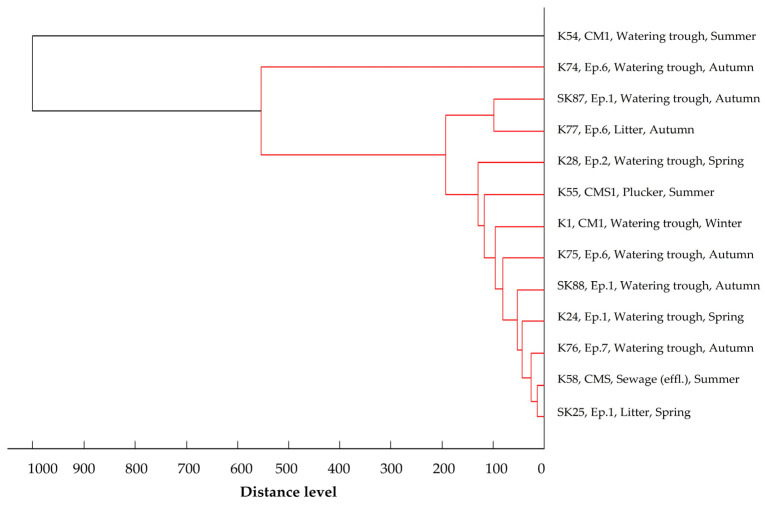
Main spectra dendrogram of *K. pneumoniae* isolates (strain I.D., origin, and season of isolation).

**Figure 7:**
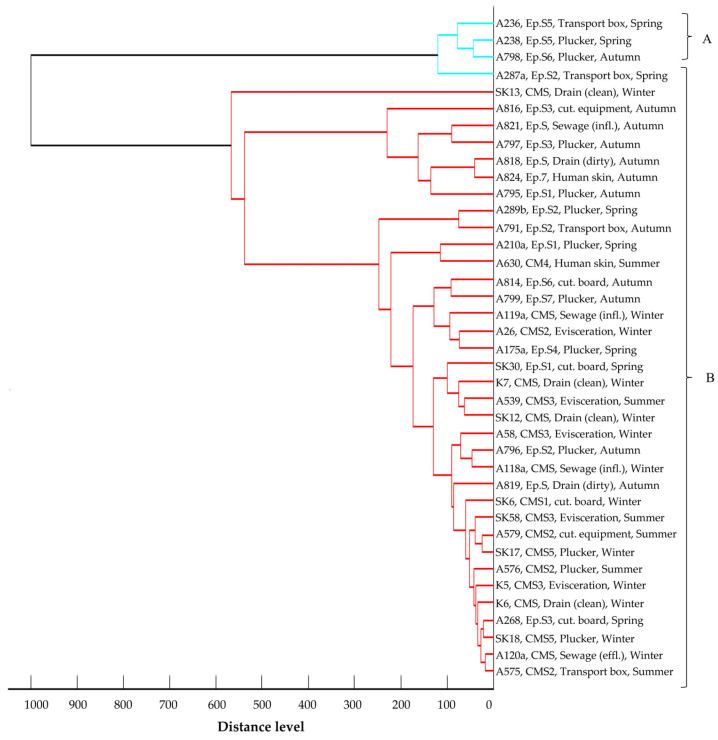
Main spectra dendrogram of *A. baumannii* isolates (strain I.D., origin, and season of isolation). The clusters are depicted by different colors in dendrogram branches and capital letters.

The main spectra dendrogram of *E. coli* isolates (Figure 5) revealed two distinct clusters when the cut-off value was set at a 400-distance level. Cluster A encompasses seven strains, three from Central Macedonia and four from Epirus, isolated from the slaughterhouse environment during summer and autumn. Cluster B comprises 80 strains isolated across all geographical regions and seasons. In the main spectra dendrogram of *K. pneumoniae* isolates (Figure 6), all isolates were organized in one cluster. Yet, a *K. pneumoniae* strain found in a watering trough on a poultry farm in Central Macedonia during the summer showed enough dissimilarity from the other isolates, resulting in it not being assigned to the recognized cluster. Similarly to *E. coli*, the main spectra dendrogram (Figure 7) of *A. baumannii* shows two clusters. Cluster A consists of four strains, all isolated from the slaughterhouse environment of the Epirus region during the spring and autumn, whereas Cluster B comprises 34 strains isolated across all geographical regions and seasons. In all three dendrograms, a close relationship is mainly observed among isolates collected within the same region and season from the same slaughterhouse or poultry farm. However, close relationships are also evident among strains isolated from different slaughterhouses and different poultry farms within the same region or even across different geographical regions. Moreover, close relationships were also observed between specific farm and slaughterhouse isolates, whereas small distance levels were also observed among strains isolated during different seasons, either from the same or different poultry farms, slaughterhouses, and geographical regions. No clear correlations were observed between the clustering of the strains and their resistance profiles or the β-lactamase genes they harbored.

### 3.5. Phylogenetic Analysis of E. coli Isolates

**Figure 8:**
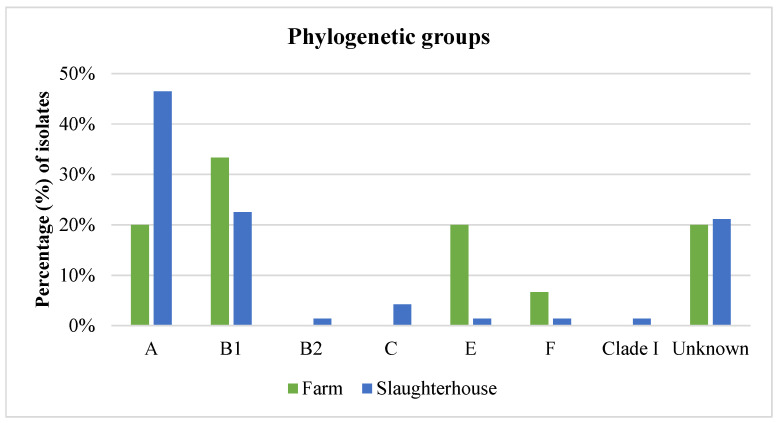
Distribution of phylogenetic groups of *E. coli* isolates in the farm and slaughterhouse environment.

In the phylogenetic analysis of the *E. coli* isolates, the majority were classified into phylogenetic groups A (41.4%, n = 36) and B1 (24.1%, n = 21). A smaller portion was assigned to phylogenetic group E (4.6%, n = 4), group C (3.4%, n = 3), group F (2.3%, n = 2), group B2 (1.1%, n = 1), and Clade I (1.1%, n = 1). Notably, no isolates were classified into group D. Additionally, in 19 strains (21.8%), including the one isolated from the poultry farmer, none of the analyzed genes were detected and were thus categorized as belonging to an unknown phylogenetic group (Figure 8, Table S8).

### 3.6. Medical and Veterinary History Results

Resistant strains were found in two poultry breeders, one residing in a major city in the Central Macedonia region, and the other in a small village in the Epirus region. Both individuals are non-smokers, with the breeder from Central Macedonia consuming little to no alcohol, while the one from Epirus consumes alcohol at a moderate level. In terms of health, the Central Macedonia breeder suffers only from hypertension, whereas the Epirus breeder is generally healthy. Neither has been recently hospitalized nor received antibiotics. However, the breeder from Epirus admitted to occasionally self-medicating with antibiotics without a doctor’s prescription or in order to treat a “non-bacterial” infection. Among the remaining ten poultry breeders from whom no resistant strains were isolated, four are current smokers (with six having smoked at least once), and only two abstain from alcohol. Furthermore, nine individuals had at least one underlying condition, one had recently been hospitalized and undergone surgery, and two had taken antibiotics shortly before the sampling period. There were no significant differences in human characteristics or antibiotic use behaviors between breeders with positive and negative test results (Tables S11–S14).

Resistant strains were identified in the environment of ten poultry farms. In seven farms, resistant strains were isolated during one season, while in three farms (two in Central Macedonia and one in Epirus), resistant strains were found in both seasons. Across all farms, vaccination programs were diligently followed, and breeding conditions were generally satisfactory. In two poultry farms where ceftazidime-resistant *E. coli* strains were detected, outbreaks of *Staphylococcus aureus* or *E. coli* had occurred, prompting the administration of antibiotics to the flock (trimethoprim–sulfodiazine or tetracycline). Additionally, in three other farms, antibiotics were administered empirically for therapeutic purposes, such as amoxicillin and cephalexin, without identifying the responsible microorganisms. Notably, two poultry farms with β-lactam-resistant strains isolated in both seasons had administered various antibiotics, including amoxicillin. In one of these farms, improper antibiotic administration practices were observed, as antibiotics were used for prophylactic and metaphylactic purposes in addition to therapy, and a stock of antibiotics, including amoxicillin, was kept on the farm. On the other hand, in five farms where resistant strains were found, no antibiotics had been administered in the three months prior to sampling. Surprisingly, in one farm with no recent antibiotic administration and excellent breeding conditions, resistant *E. coli* and *K. pneumoniae* were still isolated from the environment in both sampling seasons. In contrast, two poultry farms, where no resistant *E. coli*, *K. pneumoniae*, or *Acinetobacter* spp. strains were found, still used antibiotics, such as doxycycline and trimethoprim–sulfodiazine in their flocks. No significant differences were noted in farm characteristics and antibiotic usage between farms that harbored AMR strains and those that did not (Tables S15–S18). Regarding regional differences in ESBL *E. coli*, the only significant discrepancy between Central Macedonia and Epirus region farms was barn temperature (*p*-value = 0.02 < 0.05), with barn temperatures averaging 20–22 °C and 31 °C, respectively.

## 4. Discussion

In the present study, the prevalence rates of ESBL/AmpC *E. coli* in the farm environment are notably lower compared to those reported in other studies. Tansawai et al. [32] reported ESBL-producing *E. coli* in approximately 25% of chicken fecal samples and 25% of environmental samples (soil, water) from 27 farms in Thailand. In other studies from the Netherlands, Dierikx et al. [33] noted that within-farm prevalence of ESBL/AmpC *E. coli* was ≥80% in 22 out of 26 farms, while Huijbers et al. [34] found that 96.4% of pooled cloacal samples were positive for ESBL/AmpC *E. coli*. Moreover, high populations of ESBL *E. coli* were found in dust, slurry, and air inside the barn [6,35]. These variations in prevalence rates are primarily attributed to differences in microbial populations in the environment, influenced by geographical location, seasonal factors, and disparities in sampling techniques, sample sizes, and methodologies across studies. In contrast to the farm environment, the prevalence of ESBL/AmpC *E. coli* in environmental samples from both slaughterhouses was notably higher, aligning more closely with the findings of other European studies. In a study by von Tippelskirch et al. [36], 28% of environmental samples from a poultry slaughterhouse in Germany harbored ESBL-/AmpC-producing Enterobacteriaceae, predominantly *E. coli* isolates. Similarly, Savin et al. [37] found prevalence rates of ESBL *E. coli* in environmental samples from two slaughterhouses in Germany at 65.9% and 63.4% respectively; interestingly, ESBL *E. coli* was detected at all sampling points in both slaughterhouses. The significantly higher prevalence rates of *E. coli* in the slaughterhouse environment compared to the farm environment can be attributed to the slaughterhouse’s role as a facility where multiple flocks from different poultry farms are congregated in a single location for a short duration, facilitating the increased dissemination of bacterial species into the environment.

In contrast to ESBL *E. coli*, the prevalence of ESBL *K. pneumoniae* in farm environmental samples of the present study was slightly higher compared to the results of other international studies; Veloo et al. [38] reported that only 3 out of 104 environmental samples from poultry farms in Malaysia tested positive for ESBL *K. pneumoniae*, with two strains being isolated from soil and one from effluent water. Similarly, Li et al. [39] noted that ESBL *K. pneumoniae* isolates were detected in approximately 4% of environmental samples from poultry farms in China. Notably, most of these strains were isolated from water samples, which aligns with our findings, as a significant number of *K. pneumoniae* strains were retrieved from watering troughs. This suggests that *K. pneumoniae* growth is favored in environments with higher humidity levels, as previously reported by other studies [40,41]. However, the prevalence of ESBL *K. pneumoniae* was notably lower in environmental samples collected from slaughterhouses compared to those from poultry farms, with ESBL *K. pneumoniae* being isolated only from pluckers and effluent sewage. This finding is consistent with the results reported by Projahn et al. [42], who found ESBL *K. pneumoniae* on defeathering machines in German poultry slaughterhouses. Savin et al. [37] also noted the presence of ESBL *K. pneumoniae* in transport crates, stunning facilities, scalding water, eviscerators, and sewage (both influent and effluent) in German slaughterhouses, albeit in lower numbers compared to ESBL *E. coli*.

Concerning resistant *Acinetobacter* spp., an opposite trend to ESBL *K. pneumoniae* was observed, as most *Acinetobacter* isolates originated from the slaughterhouse environment. Veloo et al. [38] reported that only three resistant *Acinetobacter* isolates were recovered from 104 environmental samples in poultry farms in Malaysia. Similarly, Schmitz et al. [43] found only two *A. baumannii* strains among 132 boot swab samples collected from German turkey farms during rearing and before slaughter. However, both susceptible and resistant strains of *A. baumannii* were isolated at a high prevalence rate of 79.7% from chick-box papers (n = 118) of one-day-old turkey chicks. Savin et al. [37] documented very high prevalence rates of resistant *Acinetobacter* spp. of the ACB complex from two German poultry slaughterhouses (63.4% and 73.2%), isolated from all sampling points within the slaughterhouses. This aligns with the findings of this study, wherein resistant *Acinetobacter* spp. were isolated from all slaughterhouse sampling points, with higher prevalence rates observed in poultry pluckers.

Regarding the human samples, this study is probably among the first ones, to the best of our knowledge, to report the isolation of β-lactam-resistant *A. baumannii* isolates from the skin of poultry breeders. Still, the prevalence rates of resistant bacteria observed are lower compared to those reported in other studies, likely due to the absence of rectal swabs or fecal samples in the analysis, which are considered more sensitive, but they are often associated with low compliance. Tansawai et al. [32] found ESBL-producing *E. coli* in 50% of fecal samples from 32 farmers and their family members in Thailand, while 6 out of 18 poultry farmers (33%) tested positive for ESBL/AmpC β-lactamase-producing *E. coli* in their feces in the Netherlands [33]. In another study in the Netherlands, Huijbers et al. [34] stratified human samples (fecal swabs) based on the level of contact with live broilers, reporting that the overall prevalence of ESBL *E. coli* among humans was 19.1%, with rates of 14.3% among individuals with low contact and 27.1% among those with high contact with live broilers. Similarly, Wadepohl et al. [44] noted that while ESBL-producing *E. coli* was detected in 5.1% of fecal samples from slaughterhouse workers in Germany, a higher prevalence of 10% was observed in the subgroup with “higher exposure” (involved in hanging poultry and evisceration) compared to 2.9% in the subgroup with “lower exposure” (engaged in cutting and organization). The disparity in the prevalence rates of resistant strains between poultry breeders and slaughterhouse workers observed in the present study may be attributed to the use of protective equipment by the latter when handling animals, along with their adherence to more stringent hygienic practices compared to poultry breeders. These practices likely reduce their exposure to such bacteria.

Seasonal variations significantly impacted the rates of ESBL/AmpC *E. coli* in both the farm and slaughterhouse environments. This aligns with findings by Prendergast et al. [45], who observed peak recovery of resistant *Enterobacteriaceae*, predominantly *E. coli*, from Irish farm influent and effluent during summer and September, with notably fewer occurrences in winter compared to other seasons. Beyond direct environmental factors influenced by seasons such as temperature, humidity, and climate, seasonal variations also affect human activities, which correlate with fluctuating prevalence rates of resistant bacteria in the environment [46]. Prior studies have also indicated that infection rates of *E. coli* and *K. pneumoniae* peak in the summer [47,48]. Regional variations were also notable, particularly in the prevalence rates of *E. coli* in the farm environment. Although it is acknowledged that higher temperatures can accelerate the decline rate of *E. coli* in the environment [49], other unique environmental and climatic factors inherent to each region, influenced by their respective topographies, likely contributed to these regional disparities. Other studies have also noted regional disparities in the prevalence rates of resistant microorganisms within the same country. For example, Friese et al. [50] reported 100% prevalence of ESBL/AmpC *E. coli* at broiler farms in both the northwest and northeast regions of Germany, whereas Dahms et al. [51] found a 50% prevalence of ESBL/AmpC *E. coli* at broiler farms in Mecklenburg-Western Pomerania in northeast Germany. Additionally, Savin et al. [37] conducted environmental sampling at two distinct poultry slaughterhouses in Germany, revealing that 41.5% of samples from six out of seven locations within one slaughterhouse tested positive for ESBL *K. pneumoniae*, while only 7.3% of samples from the other slaughterhouse exhibited ESBL *K. pneumoniae*. These variations were attributed to differences in the content of colonized flocks supplied by various fattening farms, with the first slaughterhouse exhibiting higher slaughtering capacity compared to the other.

All *E. coli* and *K. pneumoniae* isolates, along with most *Acinetobacter* isolates, were categorized as MDR. This finding aligns with the conclusions of Prendergast et al. [45] and Veloo et al. [38], who reported that all ESBL-producing *E. coli* and *K. pneumoniae* isolates from broiler farm environments demonstrated multidrug resistance. Likewise, Li et al. [39] noted that 87.88% of ESBL *K. pneumoniae* isolates from broiler farm environments exhibited MDR traits. However, it is noteworthy that Veloo et al. [38] reported that none of the *Acinetobacter* isolates exhibited MDR, which contrasts our findings and can be attributed to the lack of an antibiotic pre-enrichment step, potentially leading to an underestimation of the prevalence of multidrug-resistant *Acinetobacter* spp. Regarding the β-lactam resistance phenotype, the results of the present study are consistent with the findings of Tansawai et al. [32], who reported that 159 out of 172 (92.4%) cefotaxime-resistant *E. coli* strains from poultry, farmers, and poultry farm environments were ESBL producers. Conversely, Xexaki et al. [52] indicated that only 13.6% and 2.7% of *E. coli* strains isolated from broiler fecal samples in Greece were ESBL and AmpC producers, respectively. Similarly, Veloo et al. [38] found that only three out of 21 *K. pneumoniae* strains from poultry farm environments were ESBL producers. As with MDR strains, the absence of a selective pre-enrichment step may lead to an underestimation of the true prevalence of ESBL-producing *E. coli* and *K. pneumoniae* strains in the environment.

Among ESBL genes, *bla*_CTX-M_ genes predominated in *E. coli*, *K. pneumoniae*, and *Acinetobacter* isolates. The predominance of *bla*_CTX-M_ genes in *E. coli* isolates from broiler farm environments and farmers was also highlighted by Tansawai et al. [32], with *bla*_CTX-M-55_ being the most prevalent variant (54.1% of strains), followed by *bla*_CTX-M-14_ (28.3%) and *bla*_CTX-M-15_ (8.8%). However, contrary findings were reported by Dahms et al. [51] and Blaak et al. [6], who noted that SHV variants predominated in *E. coli* isolates from poultry farm environments, unlike in cattle, pig, and laying hens farms where CTX-M and TEM variants were more prevalent in *E. coli* isolates. Huijbers et al. [34] and von Tippelskirch et al. [36] identified *bla*_CMY-2_ as the predominant resistance gene in *E. coli* strains isolated from farmers, broiler farms, and slaughterhouse environments, followed by *bla*_SHV-12_, *bla*_CTX-M-1_, and *bla*_TEM_ variants. Savin et al. [37], who isolated *E. coli* and *K. pneumoniae* strains from poultry slaughterhouse environments, reported *bla*_TEM_ as the most common gene in *E. coli* isolates (TEM-116, TEM-52, TEM-1, TEM-20), followed by *bla*_CTX-M_ (CTX-M-15, CTX-M-1) and *bla*_SHV_ (SHV-12, SHV-2, SHV-38). Conversely, the predominant gene in *K. pneumoniae* isolates was *bla*_SHV_ (SHV-2, SHV-27, SHV-28, SHV-1, SHV-25), followed by *bla*_CTX-M-1_ and *bla*_TEM_ variants. Similarly, Li et al. [39] observed that SHV was the predominant ESBL in *K. pneumoniae* isolates from poultry farm environments, followed by TEM and CTX-M, with approximately 40% of the strains also carrying the AmpC gene *bla*_DHA_. The geographic origin of the strains significantly correlates with the distribution of β-lactamase genes in Enterobacterales, contributing to the discrepancies observed in previous studies. Regarding *Acinetobacter* spp., most isolates in the present study did not harbor the investigated β-lactamase genes. Apart from enzymatic degradation by β-lactamases, resistance to β-lactams in Gram-negative bacteria can be attributed to various other mechanisms, such as target alteration of the penicillin-binding proteins (PBPs), decreased permeability of the β-lactam into the bacterial cell, and efflux upregulation [53]. Moreover, *Acinetobacter* isolates often carry the *Acinetobacter*-derived cephalosporinase (ADC), an AmpC cephalosporinase conferring resistance to extended-spectrum cephalosporins [54]. Savin et al. [55,56] reported that the majority of *A. baumannii* isolates from environments associated with poultry production possessed ADC variants and Ambler class A β-lactamases, such as *bla*_CTX-M-115_ and *bla*_PER-7_. Additionally, some *Acinetobacter* isolates harbored numerous OXA variants capable of hydrolyzing carbapenems, primarily *bla*_OXA-51_, *bla*_OXA-68_, *bla*_OXA-69_, and *bla*_OXA-71_, posing a significant threat to public health.

MALDI-TOF is widely recognized as a quick and user-friendly tool for analyzing bacterial strains and identifying similarities or differences among them. It serves as an alternative to other labor-intensive, time-consuming genetic methods like pulsed-field gel electrophoresis (PFGE), which require highly skilled personnel, since it has demonstrated its value in clustering and comparing resistant isolates [57]. Studies have shown strong concordance between MALDI-TOF and PFGE results for strains such as ESBL *E. coli* and carbapenem-resistant *K. pneumoniae* [57,58], further supporting its application in these analyses. Geographical origin and seasonality emerged as pivotal factors influencing the clustering of isolates, based on their protein spectra. The closest proximity was frequently observed among strains derived from samples collected from poultry farms and slaughterhouses within the same region and season. This suggests that the unique climatic and environmental conditions inherent to each geographical area and season exerted selective pressures on strains capable of surviving and persisting in poultry farm and slaughterhouse environments. Furthermore, the small distance level observed among strains from different samples within the same poultry farm or slaughterhouse hints at potential cross-contamination. However, certain strains exhibited close relatedness across different poultry farms and slaughterhouses, spanning diverse geographical regions. Given that all strains originated from environments closely linked to poultry production, it implies that these habitats share specific environmental conditions, largely influenced by poultry, which shape the selection, survival, and proliferation of microorganisms with shared traits. The identification of strains with small distance levels between poultry farms and the slaughterhouse environment suggests the likeliness of transmission of resistant strains from farms to slaughterhouses, likely introduced via poultry. Notably, human isolates showed closer relationships with strains isolated from the slaughterhouse environment rather than those from farms, emphasizing the role of human activities in disseminating resistant strains from farms to slaughterhouses and perpetuating their circulation within the poultry production chain. Additionally, some strains isolated during different seasons exhibited a high degree of similarity, highlighting the enduring presence of resistant strains in the environment. Blaak et al. [6] highlighted that approximately 60% of isolates retrieved from the farm environment shared identical genetic profiles with isolates found in poultry flocks at the same time and place, underscoring poultry flocks as primary sources of environmental contamination. Furthermore, Laube et al. [35] demonstrated the potential for fecal and airborne transmission of ESBL/AmpC-producing *E. coli* from broiler farms to the surrounding environment, showing 100% genetic similarity among isolates from slurry, animal swabs, barn air, and samples taken 50 m downwind. In contrast, Tansawai et al. [32] observed high genetic diversity among ESBL-producing *E. coli* isolates from poultry, farmers, and the environment, indicating that the presence of these isolates in backyard poultry farms may not be attributable to the proliferation of a single clone but rather to the horizontal transfer of β-lactamase genes in the poultry farm environment; nevertheless, they also noted instances of clonal transmission, with *E. coli* strains within a farm and from different farms sharing ≥ 85% genetic similarity based on their pulsotypes, suggesting the contribution of both horizontal gene transfer and clonal spread to transmission. Furthermore, close genetic relatedness was observed between isolates obtained from a farmer and chickens in the same vicinity, indicating potential transmission routes between humans and poultry. This finding aligns with other studies demonstrating the bidirectional transmission of resistant strains between humans and broilers [33,34]. Direct contact between humans and broilers appears to be a key transmission route, as individuals involved in poultry farming and slaughterhouse operations exhibit higher rates of carrying ESBL/AmpC-producing isolates in their gastrointestinal tract compared to the general population [34].

Regarding the phylogenetic analysis of *E. coli* isolates, the findings of the present study align with those of Tansawai et al. [32], reporting that most isolates from poultry, farmers, and the environment in Thailand belonged to group A (59.8%), followed by group B1 (23.9%). Similarly, Huijbers et al. [34] noted that most *E. coli* strains isolated from poultry farm workers belonged to phylogroup A (44.2%) and group D (27.9%). Conversely, Blaak et al. [6] reported that the most prevalent phylogroup in the environment of Dutch poultry farms was subgroup B1 (42%) and A (35%). Discrepancies in the predominant phylogenetic group of *E. coli* in various environments are observed not only between different countries but also within different farms and slaughterhouses within the same country. In a study conducted in Germany [37], the distribution of *E. coli* isolates varied between two poultry slaughterhouses. In one slaughterhouse, most isolates belonged to phylogroups C (34.4%) and B1 (33.3%). However, a different pattern emerged in the other slaughterhouse, where phylogroup B1 (36.6%) was predominant, followed by groups A (17.2%) and C (17.2%). Similarly, in another study by von Tippelskirch et al. [36], although most *E. coli* strains isolated from the slaughterhouse environment belonged to phylogroups B1 and F, different phylogenetic groups prevailed in the environment after the slaughter process of seven different flocks. These findings underscore how prevailing conditions, influenced by geographical region and season, can shape the selection and dominance of specific phylogenetic groups of *E. coli* in poultry and their surrounding environments.

In this study, an effort was made to delve into potential factors and behaviors contributing to the isolation of resistant strains from poultry breeders and the environment of poultry farms. Interestingly, all farmers carrying ESBL-producing *E. coli* or *A. baumannii* worked on positive-tested farms. However, analysis of the MSP dendrograms revealed somewhat high distance levels between the strains isolated from humans and those from the farm environment. Still, various misbehaviors in antibiotic use and consumption were observed across both positive- and negative-tested breeders. Regarding poultry farms, a priori antibiotic administration was noted not only in positive farms but also in many negative ones. Conversely, resistant strains were also isolated from farms where no antibiotics had been administered near the sampling seasons. This suggests either a high persistence of resistant strains in the environment or their introduction at an earlier stage, possibly through other inputs to the farm, such as day-old chicks, transport vehicles, or feed [43]. Just like in human contexts, misbehavior in antibiotic use was observed in poultry farms. Numerous studies have delved into potential risk factors contributing to the emergence and isolation of resistant strains from farm workers and the farm environment. Huijbers et al. [34] identified several key factors contributing to higher rates of ESBL *E. coli* isolation from fecal samples in poultry farm workers in the Netherlands, which included prolonged hours of contact with broilers, preexisting conditions such as diabetes or skin diseases, and sampling during the period from July to December. A recent systematic review [59] examined knowledge and practice gaps in antibiotic use in poultry across various developed and developing countries. Among the observed misbehaviors were the use of antibiotics for prevention and growth promotion, seeking antimicrobial advice from drug sellers and fellow farmers, insufficient understanding of antimicrobial residues, and inadequate awareness and implementation of withdrawal periods. Remarkably, only about half of the farmers surveyed had knowledge about AMR and its implications for poultry, human health, and the environment; moreover, farmers in developing countries exhibited a higher likelihood of knowledge gaps in antibiotic use compared to their counterparts in developed countries, consistent with the findings of Habiba et al. [60] and Kariuki et al. [61]. The lack of formal education for farmers, inadequate professional training, and limited farming experience emerged as significant factors associated with antibiotic use and understanding of AMR [60]. Additional studies with larger sample sizes are needed to further elucidate the risk factors related to antibiotic use behaviors and practices and their impact on the emergence and isolation of resistant strains from farmers and the environment.

## 5. Conclusions

This is the first study in Greece to unveil the presence and characteristics of β-lactam-resistant *K. pneumoniae* and *Acinetobacter* spp. in poultry farms, slaughterhouse environments, and among workers following selective enrichment and isolation using ceftazidime and meropenem, to the best of our knowledge. Moreover, this study represents one of the earliest investigations to uncover the knowledge and practices related to antimicrobial usage and the drivers of AMR among poultry farmers. Given that Greece ranks among the countries with the highest AMR rates in broilers and humans worldwide, a comprehensive understanding of the prevalence rates of resistant bacteria in both the environment and humans working in the poultry production chain is crucial. The poultry farm and slaughterhouse environment emerge as significant reservoirs of resistant strains of *E. coli*, *K. pneumoniae*, and *Acinetobacter* spp. These strains were consistently isolated across diverse geographical regions and throughout various seasons, showcasing a concerning multi-drug resistance profile. Particularly noteworthy is the universal presence of ESBL or AmpC production in all *E. coli* and *K. pneumoniae* isolates, accompanied by a diverse array of β-lactamase genes. Analysis of protein profiles revealed a close relatedness among strains isolated not only from the farm and slaughterhouse environments within the same regions and seasons, but also across different regions and seasons. Moreover, the questionnaires administered to poultry farmers unveiled misbehaviors and malpractices prevalent among both positive- and negative-tested farmers; still, the attribution to specific factors was not feasible. Further studies are imperative to evaluate the potential transmission risk of resistant strains or AMR genes from the farm and slaughterhouse environment to poultry meat and, subsequently, to humans.

## Figures and Tables

**Table 1:** Description of the collected samples.

	Sample	Samples per Unit per Season	Total Number of Samples
Farm environment	Watering troughs	2	40
	Cooling pads	1	20
	Ventilation fans	1	20
	Poultry litter	1	20
	Total	5	100
Slaughterhouse environment	Chicken transport boxes	5 *	20
	Chicken pluckers	5 *	20
	Evisceration machines	5 *	20
	Carcass cutting equipment	5 *	20
	Cutting boards	5 *	20
	Drains of dirty area	2	8
	Drains of clean area	2	8
	Sewage (influent)	1	4
	Sewage (effluent)	1	4
	Total	31	124
Human samples	Skin	1	40
	Oropharynx	1	40
	Total	2	80

* 1 sample per farm, during the slaughter of its animals.

## Data Availability

The original contributions presented in this study are included in the article/Supplementary Materials. Further inquiries can be directed to the corresponding author.

## Supplementary Materials

The following supporting information can be downloaded at: https://www.mdpi.com/article/10.3390/foods14020224/s1, Table S1: Timetable of sampling performed in the current study; Table S2: List of questions, regarding medical history and history of antibiotic consumption in poultry farms; Table S3: List of questions, regarding medical history and history of antibiotic consumption in humans; Table S4: Antibiotic discs used for antibiotic susceptibility with the disc diffusion method; Table S5: Primers used for molecular screening of β-lactamase genes; Table S6: Primers used for phylogenetic analysis of *E. coli* isolates; Table S7: Quadruplex PCR results and classification of *E. coli* isolates to phylogenetic groups; Table S8: Characterization of the resistant *Escherichia coli* isolates; Table S9: Characterization of the resistant *Klebsiella pneumoniae* isolates; Table S10: Characterization of the resistant *Acinetobacter baumannii* and *Acinetobacter pittii* (strain I.D.: 14 and 30) isolates; Table S11: Demographic characteristics of poultry farmers; Table S12: Characteristics and behaviors of poultry breeders (n = 12) in relation to antibiotic use; Table S13: State of health of poultry farmers; Table S14: Antibiotic use in poultry farmers; Table S15: Antibiotic use and characteristics of broiler farms (n = 10); Table S16: Characteristics of broiler farms participated in the study; Table S17: History of diseases in poultry flocks; Table S18: Antibiotic use in broiler farms.
