# Supplementary material for: Prevalence, Characterization, and Proteomic Relatedness Among β-Lactam-Resistant Bacteria Throughout the Poultry Production Chain in Greece
Source: Foods. 2025 Jan 13;14(2):224. doi: 10.3390/foods14020224 (PMC11764975; doi:10.3390/foods14020224)
Supplement: Supplementary file 1 [file foods-14-00224-s001.zip › foods-3345911-supplementary.pdf]

Table S1. Timetable of sampling performed in the current study.

Season	Sampling points
Winter 2023	Poultry farms CM1, CM2, CM3, CM4, CM5<br>Slaughterhouse CMS
Spring 2023	Poultry farms E1, E2, E3, E4, E5<br>Slaughterhouse ES
Summer 2023	Poultry farms CM1, CM2, CM3, CM4, CM5<br>Slaughterhouse CMS
Autumn 2023	Poultry farms E1, E2, E3, E6, E7<br>Slaughterhouse ES

Table S2. List of questions, regarding medical history and history of antibiotic consumption in poultry farms.

**A) General information about the flock and the poultry farm**	**B) History of consumption of antibiotics**
1. Number of birds / flock	1. List of antibiotics that were used during the last 3 months (route of administration, for how long and why they were administered)
2. Age of flock	2. List of antibiotics that were used during the rearing of the current flock (route of administration, for how long and why they were administered)
3. Type of breeding	3. What are the usual reasons for the administration of antibiotics (prophylaxis, therapy, metaphylaxis)
4. Neighboring poultry farms	4. Are withdrawal periods applicable?
5. Type of disinfection	5. Is there a stock of antibiotics in the farm? Where are they stored (room temperature, refrigerator)?
6. Temperature in barn	6. Are antibiotics used within their expiration date?
7. Ventilation / ammonia in barn	7. Are antibiotics consumed only after a vet's prescription?
8. Stocking density	8. Is a microbial culture test and antibiogram conducted before prescribing antibiotics? If yes, give more information about the isolated strain (which species, resistance profile)
9. Type of bedding	9. Does the veterinarian provide guidance on the prudent use of antibiotics?
10. Consumption of food / water	
11. Animal performances	
12. Vaccination program (for which disease, age of flock in vaccination, vaccine administration, type of vaccines)	
13. History of diseases in flock (beginning, duration, therapy, outcome)	

Table S3. List of questions, regarding medical history and history of antibiotic consumption in humans.

**A) General information**	**B) History of consumption of antibiotics**
1. Gender	1. List of antibiotics that were used during the last 3 months (route of administration, for how long and why they were administered)
2. Age	2. What are the usual reasons for the administration of antibiotics (prophylaxis, therapy, metaphylaxis)
3. Place of residence	3. Is there a stock of antibiotics in the house? Where are they stored (room temperature, refrigerator)?
4. Smoking (how much, how long)	4. Are antibiotics used within their expiration date?
5. Alcohol consumption (how much, how long)	5. How frequent and easily is the consumption of antibiotics?
6. Current health disorders / comorbidities	6. Are antibiotics consumed only after a doctor's prescription?
7. Occurrence and type of immunosuppression, time of last treatment	7. Is a microbial culture test and antibiogram conducted before prescribing antibiotics? If yes, give more information about the isolated strain (which species, resistance profile)
8. Recent vaccination status	8. Does the doctor provide guidance on the prudent use of antibiotics?
9. Concomitant therapies (drugs)	
10. Surgical procedures (type and time of conduction)	
11. Allergies	
12. Previous hospitalization (time and type of hospital unit)	

Table S4. Antibiotic discs used for antibiotic susceptibility with the disc diffusion method.

	E. coli **and** *K. pneumoniae*		*Acinetobacter* **spp.**	
**Antibiotic class**	**Antibiotic**	**Abbreviation & concentration**	**Antibiotic**	**Abbreviation & concentration**
Penicillins	Ampicillin	AM* 10 μg		
	Amoxicillin-clavulanic acid	AMC 20/10 μg		
	Ampicillin-sulbactam	SAM 10/10 μg	Ampicillin-sulbactam	SAM 10/10 μg
	Piperacillin-tazobactam	TPZ 100/10 μg	Piperacillin-tazobactam	TPZ 100/10 μg
	Ticarcillinn-clavulanic acid	TIM 75/10 μg	Ticarcillin-clavulanic acid	TIM 75/10 μg
			Piperacillin	PRL 100 μg
Cephalosporins	Cefotaxime	CTX 5 μg	Cefotaxime	CTX 5 μg
	Ceftazidime	CAZ 10 μg	Ceftazidime	CAZ 10 μg
	Cefepime	FEP 30 μg	Cefepime	FEP 30 μg
	Cefoxitin	FOX 30 μg		
Carbapenems	Meropenem	MEM 10μg	Meropenemm	MEM 10μg
	Imipenem	IPM 10 μg	Imipenem	IPM 10 μg
	Ertapenem	ETP 10 μg		
Fluoroquinolones	Ciprofloxacin	CIP 5 μg	Ciprofloxacin	CIP 5 μg
	Levofloxacin	LEV 5 μg	Levofloxacin	LEV 5 μg
Aminoglycosides	Tobramycin	TOB 10μg	Tobramycin	TOB 10μg
	Amikacin	AK 30μg	Amikacin	AK 30μg
	Gentamicin	CN 10 μg	Gentamicin	CN 10 μg
Sulfonamides	Trimethoprim-sulfamethoxazole	SXT 1.25/23.75μg	Trimethoprim-sulfamethoxazole	SXT 1.25/23.75μg
Phenicols	Chloramphenicol	C 30 μg		

Tetracyclines	Tetracycline	TE 30 µg		
	Doxycycline	DO 30 µg	Doxycycline	DO 30 µg
Macrolides	Azithromycin	AZM 15 µg		

* All antibiotic discs were procured by Oxoid Ltd (United Kingdom)

Table S5. Primers used for molecular screening of β-lactamase genes.

PCR name	Target	Sequence (5′–3′)	Product (bp)	Primer concentration
Multiplex I (ESBL)	TEM variants	For: CATTTCCGTGTCGCCCTTATTC	800	0.4 μM
		Rev: CGTTCATCCATAGTTGCCTGAC		0.4 μM
	SHV variants	For: AGCCGCTTGAGCAAATTAAAC	713	0.4 μM
		Rev: ATCCCGCAGATAAATCACCAC		0.4 μM
	OXA-1, OXA-4 and OXA-30	For: GGCACCAGATTCAACTTTCAAG	564	0.4 μM
		Rev: GACCCCAAGTTTCCTGTAAGTG		0.4 μM
Multiplex II (ESBL)	CTX-M group 1 variants	For: TTAGGAARTGTGCCGCTGYA	688	0.4 μM
		Rev: CGATATCGTTGGTGGTRCCAT		0.2 μM
	CTX-M group 2 variants	For: CGTTAACGGCACGATGAC	404	0.2 μM

PCR name	Target	Sequence (5′–3′)	Product (bp)	Primer concentration
		Rev: CGATATCGTTGGTGGTRCCAT		0.2 μM
	CTX-M group 9 variants	For: TCAAGCCTGCCGATCTGGT	561	0.4 μM
		Rev: TGATTCTCGCCGCTGAAG		0.4 μM
Simplex I (ESBL)	CTX-M groups 8 and 25	For: AACRCRCAGACGCTCTAC	326	0.4 μM
		Rev: TCGAGCCGGAASGTGTYAT		0.4 μM
Multiplex III (AmpC)	ACC-1 and ACC-2	For: CACCTCCAGCGACTTGTTAC	346	0.2 μM
		Rev: GTTAGCCAGCATCACGATCC		0.2 μM
	FOX-1 to FOX-5	For: CTACAGTGCGGGTGGTTT	162	0.5 μM
		Rev: CTATTTGCGGCCAGGTGA		0.5 μM

PCR name	Target	Sequence (5′–3′)	Product (bp)	Primer concentration
	MOX family (MOX-1, MOX-2, CMY-1, CMY-8 to CMY-11 and CMY-19)	For: GCAACAACGACAATCCATCCT	895	0.2 μM
		Rev: GGGATAGGCGTAACTCTCCCAA		0.2 μM
	DHA-1 and DHA-2	For: TGATGGCACAGCAGGATATTC	997	0.5 μM
		Rev: GCTTTGACTCTTTCGGTATTCG		0.5 μM
	CIT family (LAT-1 to LAT-3, BIL-1, CMY-2 to CMY-7, CMY-12 to CMY-18 and CMY-21 to CMY-23)	For: CGAAGAGGCAATGACCAGAC	538	0.2 μM
		Rev: ACGGACAGGGTTAGGATAGY		0.2 μM
		For: CGGTAAAGCCGATGTTGCG	683	0.2 μM

PCR name	Target	Sequence (5′–3′)	Product (bp)	Primer concentration
	EBC family (ACT-1 and MIR-1)	Rev: AGCCTAACCCCTGATACA		0.2 μM
Multiplex IV (ESBL)	GES-1 to GES-9 and GES-11	For: AGTCGGCTAGACCGGAAAG	399	0.3 μM
		Rev: TTTGTCCGTGCTCAGGAT		0.3 μM
	PER-1 and PER-3	For: GCTCCGATAATGAAAGCGT	520	0.3 μM
		Rev: TTCGGCTTGACTCGGCTGA		0.3 μM
	VEB-1 to VEB-6	For: CATTTCCCGATGCAAAGCGT	648	0.3 μM
		Rev: CGAAGTTTCTTTGGACTCTG		0.3 μM

Table S6. Primers used for phylogenetic analysis of *E. coli* isolates.

PCR name	Target	Sequence (5′-3′)	Product (bp)	Primer concentration
Quadruplex	*chuA*	For: ATGGTACCGGACGAACCAAC<br>Rev: TGCCGCCAGTACCAAAGACA	288	0.2 µM<br>0.2 µM
	yjaA	For: CAAACGTGAAGTGTCAGGAG<br>Rev: AATGCGTTCCTCAACCTGTG	211	0.2 µM<br>0.2 µM
	TspE4.C2	For: CACTATTCGTAAGGTCATCC<br>Rev: AGTTTATCGCTGCGGGTCGC	152	0.2 µM<br>0.2 µM
	arpA	For: AACGCTATTCGCCAGCTTGC<br>Rev: TCTCCCCATACCGTACGCTA	400	0.4 µM<br>0.4 µM
Group E	*arpA*	For: GATTCCATCTTGTCAAAATATGCC<br>Rev: GAAAAGAAAAAGAATTCCCAAGAG	301	0.2 µM<br>0.2 µM
Group C	*trpA*	For: AGTTTTATGCCCAGTGCGAG<br>Rev: TCTGCGCCGGTCACGCCC	219	0.2 µM<br>0.2 µM

Table S7. Quadruplex PCR results and classification of *E. coli* isolates to phylogenetic groups.

arpA	*chuA*	*yjaA*	**TspE4.C2**	**Phylogroup**
+	-	-	-	A
+	-	-	+	B1
-	+	-	-	F
-	+	+	-	B2
-	+	+	+	B2
-	+	-	+	B2
+	-	+	-	A or C*
+	+	-	-	D or E**
+	+	-	+	D or E**
+	+	+	-	E or clade I***
-	-	+	-	Clade I or II
-	476 bp product	-	-	Clade III, IV or V
-	-	-	+	Unknown
-	-	+	+	Unknown
+	-	+	+	Unknown
+	+	+	+	Unknown
-	-	-	-	Unknown

* Screen using C-specific primers. If C+, then Group C, else Group A

** Screen using E-specific primers. If E+, then Group E, else Group D

*** Screen using E-specific primers. If E+, then Group E, else clade I

Table S8. Characterization of the resistant *Escherichia coli* isolates.

Strain I.D.	Sample	Location	Season	Resistance profile	MIC Colistin (µg/mL)	Characterization of resistance profile	β-lactam resistance profile	β – lactamase genes	Phylogenetic group
E1	Poultry litter	CM1	Winter	AM, AMC, C, CAZ, CIP, CTX, FEP, LEV, SXT, TE	0.06	MDR	ESBL	CTX-M-1	B1
E2	Watering trough	CM1	Winter	AM, AMC, C, CAZ, CIP, CTX, FEP, LEV, SAM, SXT, TE	0.06	MDR	ESBL	TEM, CTX-M-1	B1
E3	Transport box	CMS1	Winter	AM, AMC, C, CAZ, CIP, CTX, FEP, LEV, SXT, TE	0.06	MDR	ESBL	CTX-M-1	B1
E4	Evisceration machine	CMS1	Winter	AM, AMC, CAZ, CTX, FEP, SAM, TIM, TPZ	0.06	MDR	ESBL	TEM, CTX-M-1	C
E5	Transport box	CMS2	Winter	AM, AMC, CAZ, CTX, FEP	0.06	MDR	ESBL	CTX-M-1	A
E6	Evisceration machine	CMS2	Winter	AM, AMC, CAZ, CTX, FEP	0.06	MDR	ESBL	CTX-M-1	E
E7α	Cutting board	CMS2	Winter	AM, AMC, C, CAZ, CTX, DO, FOX, SAM, TE, TPZ	0.06	MDR	ESBL + AmpC	-	F
E8	Drain (dirty area)	CMS	Winter	AMC, C, CAZ, CIP, CTX, FEP, LEV, SAM, SXT, TE	0.06	MDR	ESBL	TEM, CTX-M-1	B1
E9	Drain (clean area)	CMS	Winter	AMC, CAZ, CTX, FEP, SXT	0.06	MDR	ESBL	CTX-M-1	B2
E12	Poultry litter	CM2	Winter	AM, AMC, C, CAZ, CTX, FEP, SXT	0.06	MDR	ESBL	CTX-M-1	E
E16α	Poultry litter	CM4	Winter	AM, AMC, C, CAZ, CIP, CN, CTX, FEP, LEV, SAM, SXT, TE, TOB, TPZ	0.06	MDR	ESBL	TEM, CTX-M-9, CIT	F
E17	Carcass cutting equipment	CMS5	Winter	AM, AMC, C, CAZ, CIP, CTX, FEP, SXT, TPZ	0.06	MDR	ESBL	CTX-M-1	A
E18	Cutting board	CMS5	Winter	AK, AM, AZM, CAZ, CIP, CN, CTX, DO, FEP, SXT, TE, TPZ	0.06	MDR	ESBL	CTX-M-1	Unknown
E19	Sewage (influent)	CMS	Winter	AM, AMC, C, CAZ, CIP, CTX, DO, TE, TPZ	0.06	MDR	ESBL	SHV	A
E20	Sewage (effluent)	CMS	Winter	AM, C, CAZ, DO, TE	0.06	MDR	ESBL	SHV	C
E21α	Transport box	Ep.S1	Spring	AM, AMC, CAZ, CIP, CTX, TE, TPZ	0.125	MDR	ESBL	SHV	A
E24	Evisceration machine	Ep.S1	Spring	AM, AMC, AZM, CAZ, CIP, CTX, DO, FEP, SXT, TE, TPZ	0.06	MDR	ESBL	SHV	A
E25	Transport box	Ep.S4	Spring	AM, CAZ, CTX, TE	0.06	MDR	ESBL	-	A
SK34b	Evisceration machine	Ep.S4	Spring	AM, CAZ, CIP, CTX, DO, FEP, SXT, TE	0.06	MDR	ESBL	CTX-M-1	A
E26	Cutting board	Ep.S4	Spring	AM, CAZ, CIP, CTX, DO, FEP, SXT, TE	0.06	MDR	ESBL	CTX-M-1	A
SK27	Plucker	Ep.S1	Spring	AM, AMC, C, CAZ, CIP, CTX, FEP, LEV, SXT	0.06	MDR	ESBL	TEM, CTX-M-1	A

Strain I.D.	Sample	Location	Season	Resistance profile	MIC Colistin (µg/mL)	Characterization of resistance profile	β-lactam resistance profile	β – lactamase genes	Phylogenetic group
E28	Cutting board	Ep.S1	Spring	AM, AMC, CAZ, CIP, CTX, DO, FEP, LEV, SXT, TE, TPZ	0.06	MDR	ESBL	TEM, CTX-M-1	A
E29	Carcass cutting equipment	Ep.S1	Spring	AM, CAZ, CIP, CTX, DO, FEP, LEV, SXT, TE, TPZ	0.06	MDR	ESBL	TEM, CTX-M-1	A
E30	Drain (dirty area)	Ep.S	Spring	AM, AMC, CAZ, CIP, CTX, DO, LEV, SXT, TE, TIM, TPZ	0.06	MDR	ESBL	-	Unknown
E31	Drain (clean area)	Ep.S	Spring	AM, CAZ, CIP, CTX, DO, FEP, SXT, TE	0.06	MDR	ESBL	CTX-M-1	A
E33	Plucker	Ep.S5	Spring	AM, AMC, C, CAZ, CIP, CTX, FEP, LEV, SXT	0.06	MDR	ESBL	TEM, CTX-M-1	A
E34	Transport box	Ep.S5	Spring	AM, AMC, C, CAZ, CIP, CN, CTX, DO, FEP, LEV, SXT, TE, TOB	0.06	MDR	ESBL	TEM, CTX-M-1	B1
E35	Watering trough	Ep.3	Spring	AM, AMC, AZM, C, CAZ, CIP, CTX, FEP, LEV, SXT, TE, TOB, TPZ	0.06	MDR	ESBL	CTX-M-1	B1
E36	Poultry litter	Ep.3	Spring	AM, AMC, C, CAZ, CIP, CTX, FEP, LEV, SAM, SXT, TE, TPZ	0.06	MDR	ESBL	CTX-M-1	Unknown
E37	Plucker	Ep.S3	Spring	AM, AMC, C, CAZ, CIP, CTX, FEP, LEV, SXT, TE, TPZ	0.06	MDR	ESBL	CTX-M-1	B1
E38	Evisceration machine	Ep.S3	Spring	AM, AMC, CAZ, CTX, DO, FEP, SXT, TE, TPZ	0.06	MDR	ESBL	CTX-M-1	A
E39	Carcass cutting equipment	Ep.S3	Spring	AM, AMC, C, CAZ, CIP, CTX, FEP, LEV, TPZ	0.125	MDR	ESBL	TEM, CTX-M-1	A
E40	Cutting board	Ep.S3	Spring	AM, AMC, C, CAZ, CIP, CTX, FEP, LEV, SXT, TPZ	0.06	MDR	ESBL	TEM, CTX-M-1	A
E42	Plucker	Ep.S2	Spring	AM, AMC, C, CAZ, CIP, CTX, FEP, LEV, SXT, TE	0.06	MDR	ESBL	CTX-M-1	A
E43	Transport box	Ep.S2	Spring	AM, AMC, C, CAZ, CIP, CTX, FEP, LEV, SXT, TE, TPZ	0.06	MDR	ESBL	-	A
E45	Drain (dirty area)	Ep.S	Spring	AM, AMC, AZM, C, CAZ, CIP, CN, CTX, DO, FEP, LEV, SXT, TE, TOB	0.06	MDR	ESBL	TEM, CTX-M-1	B1
E46	Drain (clean area)	Ep.S	Spring	AM, CAZ, CIP, CTX, DO, FEP, SXT, TE, TPZ	0.06	MDR	ESBL	TEM, CTX-M-1	Unknown
E47α	Sewage (influent)	Ep.S	Spring	AM, AMC, CAZ, CIP, CTX, DO, LEV, SXT, TE, TPZ	0.06	MDR	ESBL	-	Unknown
E48	Sewage (effluent)	Ep.S	Spring	AM, AMC, AZM, C, CAZ, CIP, CN, CTX, DO, FEP, LEV, SAM, SXT, TE, TIM, TPZ	0.06	MDR	ESBL	TEM, CTX-M-1	A
E73	Watering trough	CM1	Summer	AM, AMC, C, CAZ, CIP, CTX, FEP, LEV, SXT, TE, TIM, TPZ	0.06	MDR	ESBL	TEM	A
E74	Watering trough	CM1	Summer	AM, AMC, CAZ, CIP, CTX, DO, FEP, LEV, SAM, SXT, TE, TPZ	0.06	MDR	ESBL	TEM, CTX-M-1	E
E75	Poultry litter	CM1	Summer	AM, AMC, CAZ, CIP, CTX, DO, FEP, LEV, SAM, SXT, TE, TIM, TPZ	0.06	MDR	ESBL	TEM, CTX-M-1	E
E76	Transport box	CMS1	Summer	AM, AMC, C, CAZ, CIP, CTX, FEP, LEV, SXT, TE, TPZ	0.06	MDR	ESBL	CTX-M-1	B1

Strain I.D.	Sample	Location	Season	Resistance profile	MIC Colistin (µg/mL)	Characterization of resistance profile	β-lactam resistance profile	β – lactamase genes	Phylogenetic group
E77	Evisceration machine	CMS1	Summer	AM, AMC, C, CAZ, CIP, CTX, FEP, LEV, SXT, TE, TPZ	0.06	MDR	ESBL	CTX-M-1	B1
E78	Carcass cutting equipment	CMS1	Summer	AM, AMC, CAZ, CTX, FEP, TE, TPZ	0.06	MDR	ESBL	SHV	A
E79	Cutting board	CMS1	Summer	AM, AMC, CAZ, CTX, FEP, TE	0.125	MDR	ESBL	SHV	A
E82	Watering trough	CM3	Summer	AM, AMC, C, CAZ, CIP, CTX, FEP, LEV, SXT, TPZ	0.06	MDR	ESBL	TEM, CTX-M-1	Unknown
E83	Watering trough	CM3	Summer	AM, AMC, C, CAZ, CIP, CTX, FEP, LEV, SAM, SXT, TPZ	0.06	MDR	ESBL	TEM, CTX-M-1	B1
E84	Cooling pad	CM3	Summer	AM, AMC, C, CAZ, CIP, CTX, DO, FEP, LEV, SAM, SXT, TE, TIM, TPZ	0.125	MDR	ESBL	TEM, CTX-M-1	A
E85	Transport box	CMS3	Summer	AM, AMC, C, CAZ, CTX, DO, FEP, SXT, TE	0.06	MDR	ESBL	CTX-M-1	B1
E86	Carcass cutting equipment	CMS3	Summer	AM, AMC, CAZ, CIP, CTX, FEP, TPZ	0.125	MDR	ESBL	CTX-M-1	Unknown
E87	Cutting board	CMS3	Summer	AM, AMC, CAZ, CIP, CTX, FEP, LEV, SAM, SXT, TPZ	0.06	MDR	ESBL	TEM, CTX-M-1	Unknown
E88	Drain (clean area)	CMS	Summer	AM, AMC, AZM, C, CAZ, CTX, DO, FEP, SXT, TE, TPZ	0.06	MDR	ESBL	CTX-M-1	B1
E89	Evisceration machine	CMS5	Summer	AM, AMC, AZM, C, CAZ, CTX, DO, FEP, SXT, TE, TPZ	0.06	MDR	ESBL	CTX-M-1	B1
E90	Carcass cutting equipment	CMS5	Summer	AM, AMC, AZM, C, CAZ, CIP, CTX, DO, FEP, LEV, SAM, SXT, TE, TPZ	0.06	MDR	ESBL	-	Unknown
E91	Cutting board	CMS5	Summer	AM, AMC, C, CAZ, CIP, CTX, DO, FEP, LEV, SXT, TE, TPZ	0.06	MDR	ESBL	CTX-M-1	B1
E98	Transport box	CMS2	Summer	AM, AMC, AZM, C, CAZ, CIP, CTX, FEP, LEV, SXT, TE, TPZ	0.06	MDR	ESBL	CTX-M-1	B1
E99	Plucker	CMS2	Summer	AM, AMC, C, CAZ, CIP, CTX, FEP, LEV, SXT, TE, TPZ	0.125	MDR	ESBL	CTX-M-1	Unknown
E100	Carcass cutting equipment	CMS2	Summer	AM, AMC, C, CAZ, CIP, CTX, FEP, LEV, SAM, SXT, TE, TIM, TPZ	0.06	MDR	ESBL	TEM	Unknown
E101	Cutting board	CMS2	Summer	AM, AMC, C, CAZ, CIP, CTX, DO, FEP, LEV, SXT, TE, TPZ	0.125	MDR	ESBL	CTX-M-1, FOX	B1
E105	Poultry litter	CM5	Summer	AK, AM, AMC, AZM, C, CAZ, CIP, CN, CTX, DO, FEP, LEV, SXT, TE, TPZ	0.06	MDR	ESBL	TEM, CTX-M-1	B1
E107	Plucker	CMS4	Summer	AK, AM, AMC, AZM, CAZ, CN, CTX, FEP, TPZ	0.06	MDR	ESBL	CTX-M-1	B1
E108	Carcass cutting equipment	CMS4	Summer	AK, AM, AZM, CAZ, CIP, CN, CTX, FEP, LEV, TPZ	0.06	MDR	ESBL	-	CLADE I
E109	Cutting board	CMS4	Summer	AK, AM, AZM, CAZ, CIP, CN, CTX, TPZ	0.06	MDR	ESBL	-	Unknown
E110	Sewage (influent)	CMS	Summer	AK, AM, AMC, AZM, CAZ, CIP, CN, CTX, FEP, TOB, TPZ	0.06	MDR	ESBL	-	Unknown

Strain I.D.	Sample	Location	Season	Resistance profile	MIC Colistin (μg/mL)	Characterization of resistance profile	β-lactam resistance profile	β – lactamase genes	Phylogenetic group
E111b	Sewage (effluent)	CMS	Summer	AM, AMC, AZM, CAZ, CIP, CN, CTX, FEP, LEV, TPZ	0.25	MDR	ESBL	CTX-M-1	C
E112	Watering trough	CM4	Summer	AK, AM, AMC, AZM, C, CAZ, CIP, CTX, DO, FEP, SAM, SXT, TE, TIM, TPZ	0.06	MDR	ESBL	TEM, CTX-M-1	Unknown
E113	Poultry litter	CM4	Summer	AK, AM, AMC, AZM, CAZ, CIP, CN, CTX, FEP, SXT, TE, TOB, TPZ	0.06	MDR	ESBL	TEM, SHV	A
E114	Skin (breeder)	CM4	Summer	AM, AMC, AZM, CAZ, CIP, CN, CTX, DO, FEP, LEV, TE, TIM, TPZ	0.125	MDR	ESBL	-	Unknown
E125	Transport box	Ep.S2	Autumn	AM, AMC, C, CAZ, CIP, CTX, FEP, LEV, SXT, TE, TPZ	0.125	MDR	ESBL	CTX-M-1	B1
E126	Transport box	Ep.S3	Autumn	AM, AMC, AZM, C, CAZ, CIP, CTX, FEP, LEV, SAM, SXT, TE, TPZ	0.125	MDR	ESBL	TEM	Unknown
E127	Plucker	Ep.S1	Autumn	AM, AMC, AZM, C, CAZ, CIP, CTX, DO, LEV, SAM, SXT, TE, TIM, TPZ	0.06	MDR	ESBL	TEM, SHV	B1
E128	Plucker	Ep.S2	Autumn	AM, AMC, AZM, CAZ, CIP, CTX, DO, LEV, TE	0.06	MDR	ESBL	-	Unknown
E129	Plucker	Ep.S3	Autumn	AM, AMC, CAZ, CIP, CN, CTX, FEP, TE, TPZ	0.06	MDR	ESBL	SHV	A
E130	Plucker	Ep.S7	Autumn	AM, AMC, AZM, CAZ, CIP, CN, CTX, LEV, TOB, TPZ	0.06	MDR	ESBL	-	Unknown
E131	Evisceration machine	Ep.S1	Autumn	AK, AM, AMC, CAZ, CIP, CN, CTX, DO, FEP, SXT, TE, TPZ	0.06	MDR	ESBL	TEM, CTX-M-1	A
E132	Evisceration machine	Ep.S2	Autumn	AM, AMC, C, CAZ, CIP, CN, CTX, FEP, LEV, SXT, TIM, TPZ	0.125	MDR	ESBL	TEM, CTX-M-1	A
E133	Evisceration machine	Ep.S6	Autumn	AM, CAZ, CTX, DO, FEP, SXT, TE	0.5	MDR	ESBL	-	A
E134	Evisceration machine	Ep.S7	Autumn	AM, AZM, CAZ, CIP, CTX, DO, FEP, SXT, TE, TPZ	0.125	MDR	ESBL	TEM, CTX-M-1	A
E135	Carcass cutting equipment	Ep.S3	Autumn	AM, AZM, CAZ, CIP, CN, CTX, DO, FEP, SXT, TE, TPZ	0.06	MDR	ESBL	-	A
E136	Carcass cutting equipment	Ep.S6	Autumn	AK, AM, AMC, AZM, CAZ, CIP, CN, CTX, DO, FEP, SXT, TE, TPZ	0.125	MDR	ESBL	TEM, CTX-M-1	A
E137	Cutting board	Ep.S1	Autumn	AM, AMC, C, CAZ, CIP, CTX, FEP, LEV, SXT	0.25	MDR	ESBL	TEM, CTX-M-1	A
E138	Drain (dirty area)	Ep.S	Autumn	AK, AM, AMC, AZM, CAZ, CIP, CTX, FEP, TE, TIM, TPZ	0.125	MDR	ESBL	SHV	A
E139	Drain (clean area)	Ep.S	Autumn	AM, AZM, CAZ, CIP, CTX, DO, FEP, LEV, SXT, TE, TPZ	0.06	MDR	ESBL	TEM, CTX-M-1	A
E140	Drain (clean area)	Ep.S	Autumn	AM, AMC, AZM, C, CAZ, CIP, CTX, FEP, LEV, SXT, TPZ	0.06	MDR	ESBL	TEM, CTX-M-1	A
E141	Sewage (influent)	Ep.S	Autumn	AM, CAZ, CIP, CTX	0.06	MDR	ESBL	SHV	A
E142	Sewage (effluent)	Ep.S	Autumn	AM, AMC, AZM, CAZ, CIP, CN, CTX, DO, FEP, SAM, TE, TIM, TOB, TPZ	0.125	MDR	ESBL	-	Unknown

Table S9. Characterization of the resistant *Klebsiella pneumoniae* isolates.

Strain I.D.	Sample	Location	Season	Resistance profile	MIC Colistin (μg/mL)	Characterization of resistance profile	β-lactam resistance profile	β – lactamase genes
K1	Watering trough	CM1	Winter	AM, AMC, AZM, CAZ, CIP, CTX, DO, FEP, TE, TPZ	0.125	MDR	ESBL	SHV, CTX-M-1
SK25	Poultry litter	Ep.1	Spring	AM, AMC, AZM, CAZ, CIP, CTX, DO, FEP, LEV, SAM, SXT, TE, TIM, TPZ	0.125	MDR	ESBL	TEM, SHV, CTX-M-1
K24	Watering trough	Ep.1	Spring	AK, AM, AMC, AZM, CAZ, CIP, TPZ, CTX, DO, FEP, LEV, SAM, SXT, TE, TIM	0.125	MDR	ESBL	TEM, SHV, CTX-M-1
K28	Watering trough	Ep.2	Spring	AM, AMC, AZM, CAZ, CIP, CTX, DO, FEP, LEV, SXT, TE, TIM, TPZ	0.125	MDR	ESBL	TEM, SHV, CTX-M-1
K54	Watering trough	CM1	Summer	AM, AMC, AZM, CAZ, CIP, CTX, FEP, SXT, TPZ	0.06	MDR	ESBL	CTX-M-1
K55	Plucker	CMS1	Summer	AM, AMC, AZM, CAZ, CIP, CTX, FEP, LEV, SXT, TIM, TPZ	0.06	MDR	ESBL	TEM, SHV, CTX-M-1
K58	Sewage (effluent)	CMS	Summer	AM, AMC, AZM, CAZ, CIP, CN, CTX, FEP, LEV, SAM, SXT, TIM, TPZ	0.06	MDR	ESBL	TEM, SHV, CTX-M-1
K74	Watering trough	Ep.6	Autumn	AK, AM, AMC, AZM, CAZ, CIP, CTX, DO, FEP, LEV, SAM, SXT, TE, TIM, TOB, TPZ	0.06	MDR	ESBL	TEM, OXA-1, CTX-M-1
K75	Watering trough	Ep.6	Autumn	AK, AM, AMC, AZM, CAZ, CIP, CTX, DO, FEP, LEV, SAM, SXT, TE, TIM, TOB, TPZ	0.06	MDR	ESBL	OXA-1, CTX-M-1
K76	Watering trough	Ep.7	Autumn	AM, AMC, AZM, C, CAZ, CIP, CTX, DO, FEP, LEV, SAM, SXT, TE, TIM, TOB, TPZ	0.125	MDR	ESBL	TEM, SHV, CTX-M-1
K77	Poultry litter	Ep.6	Autumn	AK, AM, AMC, AZM, CAZ, CIP, CN, CTX, DO, FEP, LEV, SAM, SXT, TE, TIM, TOB, TPZ	0.06	MDR	ESBL	OXA-1, CTX-M-1
SK87	Watering trough	Ep.1	Autumn	AM, AMC, AZM, CAZ, CIP, CTX, DO, FEP, LEV, SAM, SXT, TE, TIM, TPZ	0.06	MDR	ESBL	SHV, CTX-M-1
SK88	Watering trough	Ep.1	Autumn	AM, AMC, AZM, CAZ, CIP, CTX, FEP, LEV, TPZ	0.06	MDR	ESBL	SHV

Table S10. Characterization of the resistant *Acinetobacter baumannii* and *Acinetobacter pittii* (strain I.D.: 14 and 30) isolates.

Strain I.D.	Sample	Location	Season	Resistance profile	MIC Colistin (μg/mL)	Characterization of resistance profile	β – lactamase genes
SK6	Cutting board	CMS1	Winter	CAZ, CTX, PRL, TPZ	0.125	MDR	CTX-M-1
A26α	Evisceration machine	CMS2	Winter	CAZ, CTX, PRL, TPZ	0.5	MDR	CTX-M-1
SK12	Drain (clean area)	CMS	Winter	CAZ, CIP, CTX, PRL, TPZ	0.125	MDR	CTX-M-1, CTX-M-9
SK13	Drain (clean area)	CMS	Winter	CAZ, CIP, CTX, DO, LEV, PRL, TPZ	0.125	MDR	-
A58	Evisceration machine	CMS3	Winter	CAZ, CTX, PRL, SXT, TPZ	0.25	MDR	-
K5	Evisceration machine	CMS3	Winter	CAZ, CTX, PRL, SXT, TPZ	0.125	MDR	-
K6	Drain (clean area)	CMS	Winter	CAZ, CTX, PRL, TPZ	0.125	MDR	-
K7	Drain (clean area)	CMS	Winter	CAZ, CTX, PRL, TPZ	0.06	MDR	-
SK17	Plucker	CMS5	Winter	CAZ, CTX, PRL, TPZ	0.25	MDR	CTX-M-1, CTX-M-9
SK18	Plucker	CMS5	Winter	CAZ, CTX, PRL	0.5	-	-
A118α	Sewage (influent)	CMS	Winter	CAZ, CTX, PRL, TPZ	0.125	MDR	-
A119α	Sewage (influent)	CMS	Winter	CAZ, CN, CTX, FEP, PRL, SXT, TPZ	0.25	MDR	-
A120α	Sewage (effluent)	CMS	Winter	CAZ, CTX, PRL, TPZ	0.125	MDR	-
SK33	Transport box	Ep.S4	Spring	CAZ, CIP, CTX, LEV, PRL, SXT	0.06	MDR	-
SK30	Cutting board	Ep.S1	Spring	CAZ, CIP, CTX, DO, FEP, LEV, PRL, SAM, SXT, TIM	0.125	MDR	CTX-M-1
A175a	Plucker	Ep.S4	Spring	CAZ, CTX, PRL	0.25	-	CTX-M-1
A210α	Plucker	Ep.S1	Spring	CAZ, CTX, PRL, SXT, TPZ	0.25	MDR	-
A236	Transport box	Ep.S5	Spring	CAZ, CTX, PRL, TPZ	0.5	MDR	-
A238	Plucker	Ep.S5	Spring	CAZ, CTX, PRL, TPZ	0.25	MDR	CTX-M-1, CTX-M-9
A268	Cutting board	Ep.S3	Spring	CAZ, CTX, PRL, TPZ	0.125	MDR	-
A289b	Plucker	Ep.S2	Spring	AK, CAZ, CN, CTX, FEP, PRL, SXT	0.25	MDR	-
A287α	Transport box	Ep.S2	Spring	CAZ, CTX, PRL	0.5	-	-
A539	Evisceration machine	CMS3	Summer	CAZ, CTX, PRL, TPZ	0.25	MDR	CTX-M-1
SK58	Evisceration machine	CMS3	Summer	CAZ, CTX, PRL, TPZ	0.25	MDR	-
A575	Transport box	CMS2	Summer	CAZ, CTX, PRL, SXT, TPZ	0.125	MDR	-

Strain I.D.	Sample	Location	Season	Resistance profile	MIC Colistin (μg/mL)	Characterization of resistance profile	β – lactamase genes
A576	Plucker	CMS2	Summer	CAZ, CN, CTX, PRL, TPZ	0.5	MDR	-
A579	Carcass cutting equipment	CMS2	Summer	AK, CAZ, CN, CTX, FEP, PRL, SXT, TPZ	0.06	MDR	-
A630	Skin (breeder)	CM4	Summer	CAZ, CTX, PRL	0.25	-	-
A824	Skin (breeder)	Ep.7	Autumn	CAZ, CTX, PRL, TPZ	0.125	MDR	-
A779	Ventilation fan	Ep.7	Autumn	AK, CAZ, CIP, CN, CTX, FEP, LEV, PRL, SXT, TIM, TOB, TPZ	0.06	MDR	-
A791	Transport box	Ep.S2	Autumn	AK, CAZ, CIP, CN, CTX, FEP, LEV, PRL, SXT, TIM, TOB, TPZ	0.25	MDR	-
A795	Plucker	Ep.S1	Autumn	CAZ, CTX, PRL, TPZ	0.125	MDR	-
A796	Plucker	Ep.S2	Autumn	CAZ, CTX, PRL, SXT, TPZ	0.125	MDR	CTX-M-1, CTX-M-9
A797	Plucker	Ep.S3	Autumn	CAZ, CTX, PRL, SXT, TPZ	0.125	MDR	-
A798	Plucker	Ep.S6	Autumn	CAZ, CTX, PRL	0.06	-	CTX-M-1, CTX-M-9
A799	Plucker	Ep.S7	Autumn	CAZ, CTX, PRL, SXT, TPZ	0.06	MDR	-
A814	Cutting board	Ep.S6	Autumn	CAZ, CTX, PRL, TPZ	0.06	MDR	-
A816	Carcass cutting equipment	Ep.S3	Autumn	CAZ, CTX, PRL, TPZ	0.125	MDR	-
A818	Drain (dirty area)	Ep.S	Autumn	CAZ, CTX, PRL, TPZ	0.06	MDR	-
A819	Drain (dirty area)	Ep.S	Autumn	CAZ, CTX, PRL, TPZ	0.06	MDR	-
A821	Sewage (influent)	Ep.S	Autumn	AK, CAZ, CN, CTX, FEP, PRL, SXT, TPZ	0.06	MDR	-

* *Acinetobacter pittii*

Table S11. Demographic characteristics of poultry farmers.

Human I.D.	Farm	Gender	Age	Place of residence	Smoking (moment of speaking)	Pack years	Alcohol consumption (units/week)
H1	CM1	Male	62	Sindos (Town)	Yes	63	0
H2	CM2	Male	40	Thessaloniki (City)	Yes	29	5
H3	CM3	Male	57	Vathilakkos (Town)	Yes	37	4
H4	CM4	Male	65	Thessaloniki (City)	No	0	0
H5	CM5	Male	55	Lakkia (Village)	No	0	20
H6	Ep.1	Male	66	Ioannina (City)	No	19	17.5
H7	Ep.2	Male	44	Ag. Ioannis (Village)	No	40	0.625
H8	Ep.3	Male	50	Ag. Ioannis (Village)	Yes	33	1.25
H9	Ep.4	Male	39	Neohori (Village)	No	0	17.5
H10	Ep.5	Female	51	Pedini (Village)	No	0	2.5
H11	Ep.6	Female	67	Koutselio (Village)	No	0	0
H12	Ep.7	Male	42	Kosmera (Village)	No	0	6.25

Table S12. Characteristics and behaviors of poultry breeders (n=12) in relation to antibiotic use.

Variable	Total Number (%)	p-value
**Gender**		
Male	10 (83.3%)	1.0
Female	2 (16.7%)	
**Age**		
≤40	2 (16.7%)	0.653
41-64	7 (58.3%)	
≥65	3 (25%)	
**Smoking** (moment of speaking)		
Yes	4 (33.3%)	0.495
No	8 (66.7%)	
**Pack years**		
0	6 (50%)	
1-19	1 (8.3%)	0.579
20-39	3 (25%)	
≥40	2 (16.7%)	
**Alcohol consumption** (units/week)		
0	3 (25%)	
0.1-2.5	3 (25%)	1.0
2.6-10	3 (25%)	
≥10.1	3 (25%)	
**Current health disorders / comorbidities**		
Yes	10 (83.3%)	0.284
No	2 (16.7%)	
**Immunosuppression**		
Yes	0 (0%)	-
No	12 (100%)	
**Recent vaccinations** (e.g. Covid-19, Influenza, Pneumococcus etc.)		
Yes	12 (100%)	-
No	0 (0%)	
**Concomitant therapies**		
Yes	9 (75%)	0.521
No	3 (25%)	
**Recent surgical procedures**		
Yes	1 (8.3%)	1.0
No	11 (91.7%)	
**Allergies**		
Yes	2 (16.7%)	1.0
No	10 (83.3%)	
**Previous hospitalization**		
Yes	1 (8.3%)	1.0
No	11 (91.7%)	
**Recent antibiotic consumption**		
Winter-Spring		
Yes	0 (0%)	
No	10 (100%)	1.0
Summer-Autumn		
Yes	2 (20%)	
No	8 (80%)	

Variable	Total Number (%)	p-value
**Reasons for antibiotic consumption**		
Treatment	12 (100%)	1.0
Prophylaxis	2 (16.7%)	
Metaphylaxis	0 (0%)	
**Stock of antibiotics in house**		
Yes	4 (33.3%)	0.521
No	8 (66.7%)	
**Use of antibiotics before their expiration**		
Yes	12 (100%)	-
No	0 (0%)	
**Frequency and easiness of antibiotic consumption**		
Never (only when absolutely necessary)	7 (58.3%)	
Rare	5 (41.7%)	1.0
Sometimes	0 (0%)	
Very often	0 (0%)	
**Antibiotic consumption after doctor's prescription**		
Always	11 (91.7%)	
Usually	1 (8.3%)	0.1
Sometimes	0 (0%)	
Rarely	0 (0%)	
**Microbial culture test and antibiogram performed**		
Always	0 (0%)	
Usually	0 (0%)	-
Sometimes	0 (0%)	
Never	12 (100%)	
**Guidance by doctor**		
Complete	0 (0%)	1.0
Partial	11 (91.7%)	
None	1 (8.3%)	

Table S13. State of health of poultry farmers.

Human I.D.	Farm	Current health disorders / comorbidities	Immunosuppression	Vaccination	Concomitant therapies	Surgical procedures	Allergies	Previous hospitalization
H1	CM1	No	No	Covid-19	No	Knee arthroplasty in 2021	No	Yes (for knee arthroplasty)
H2	CM2	Diverticula	No	Covid-19	No	No	No	No
H3	CM3	Hypertension	No	Covid-19, Influenza, Pneumococcus	Amlodipine	No	No	No
H4	CM4	Hypertension	No	Covid-19	Amlodipine	No	No	No
H5	CM5	Asthma	No	Covid-19	Budesonide / Formoterol Fumarate	No	Bee sting	No
H6	Ep.1	Hypertension, Asthma, Hypothyroidism	No	Covid-19, Influenza, Pneumococcus	Valsartan, Salbutamol, Levothyroxine	No	Allergic asthma	No
H7	Ep.2	Diabetes type II	No	Covid-19, Influenza	Insulin	No	No	No
H8	Ep.3	Hypercholesterolemia	No	Covid-19	Atorvastatin	No	No	No
H9	Ep.4	Hypercholesterolemia	No	Covid-19	No	No	No	No
H10	Ep.5	Hypercholesterolemia, Hashimoto	No	Covid-19, Influenza	Levothyroxine	No	No	No
H11	Ep.6	Hypercholesterolemia	No	Covid-19, Influenza, Pneumococcus	Aspirin, Atorvastatin	No	No	No
H12	Ep.7	No	No	Covid-19	No	No	No	No

Table S14. Antibiotic use in poultry farmers.

Human I.D.	Farm	Consumed antibiotics	Reasons for antibiotic consumption	Stock of antibiotics in house	Use of antibiotics before their expiration	Frequency and easiness of antibiotic consumption	Antibiotic consumption after doctor's prescription	Microbial culture test and antibiogram performed	Guidance by doctor
H1	CM1	No	Treatment	No	Yes	Never (only when absolutely necessary)	Always	Never	Partial
H2	CM2	Ciprofloxacin and Metronidazole for Diverticula (summer only)	Treatment	Yes (refrigerator)	Yes	Rare	Always	Never	Partial
H3	CM3	No	Treatment and prophylaxis	No	Yes	Rare	Always	Never	No
H4	CM4	No	Treatment	No	Yes	Never (only when absolutely necessary)	Always	Never	Partial
H5	CM5	No	Treatment	No	Yes	Never (only when absolutely necessary)	Always	Never	Partial
H6	Ep.1	No	Treatment	No	Yes	Never (only when absolutely necessary)	Always	Never	Partial
H7	Ep.2	Amoxicillin for toothache (spring only)	Treatment	Yes (room temperature)	Yes	Never (only when absolutely necessary)	Always	Never	Partial
H8	Ep.3	No	Treatment	Yes (room temperature)	Yes	Rare	Always	Never	Partial
H9	Ep.4	No	Treatment	Yes (refrigerator)	Yes	Never (only when absolutely necessary)	Always	Never	Partial
H10	Ep.5	No	Treatment	No	Yes	Never (only when absolutely necessary)	Always	Never	Partial
H11	Ep.6	No	Treatment and prophylaxis	No	Yes	Rare	Always	Never	Partial
H12	Ep.7	No	Treatment	No	Yes	Rare	Usually	Never	Partial

Table S15. Antibiotic use and characteristics of broiler farms (n=10).

Variable	Total Number (%)		p-value
	Winter-Spring	Summer-Autumn	
**Number of birds / flock**			
≤ 20000	3 (30%)	3 (30%)	
20001 - 40000	2 (20%)	3 (30%)	0.479
40001 - 60000	3 (30%)	2 (20%)	
≥ 60001	2 (20%)	2 (20%)	
**Type of breeding**			-
Intensive	10 (100%)	10 (100%)	
Organic	0 (0%)	0 (0%)	
Other	0 (0%)	0 (0%)	
**Age of flock**			
≤40	0 (0%)	2 (20%)	0.523
41-44	5 (50%)	6 (60%)	
≥45	5 (50%)	2 (20%)	
**Neighboring poultry farms**			
Yes	7 (70%)	5 (50%)	0.642
No	3 (30%)	5 (50%)	
**Disinfection**			
Yes	10 (100%)	10 (100%)	-
No	0 (0%)	0 (0%)	
**Temperature in barn**			
20-22° C	5 (50%)	5 (50%)	1.0
31° C	5 (50%)	5 (50%)	
**Ventilation / ammonia in barn**			
Good / low	8 (80%)	10 (100%)	0.589
Mediocre / mediocre	1 (10%)	0 (0%)	
Not good / high	1 (10%)	0 (0%)	
**Stocking density (birds/m^2)**			
13	1 (10%)	1 (10%)	1.0
14	2 (20%)	3 (30%)	
15	2 (20%)	1 (10%)	
No data	5 (50%)	5 (50%)	
**Type of bedding**			
Rice hull	7 (70%)	4 (40%)	
Sawdust	1 (10%)	2 (20%)	0.184
Straw	2 (20%)	4 (40%)	
Other	0 (0%)	0 (0%)	
**Consumption of food / water**			
≤ 100000	5 (50%)	5 (50%)	0.275
100001 - 200000	3 (30%)	3 (30%)	
≥ 200001	2 (20%)	2 (20%)	
**Animal performances**			
Excellent	2 (20%)	2 (20%)	0.530
Good	4 (40%)	8 (80%)	
Mediocre	4 (40%)	0 (0%)	
Not good	0 (0%)	0 (0%)	
**Disease in flock**			0.521
Yes	0 (0%)	2 (20%)	

Variable	Total Number (%)		p-value
	Winter-Spring	Summer-Autumn	
No	10 (100%)	8 (80%)	
**Antibiotic consumption (3 months in advance)**			
Yes	2 (20%)	0 (0%)	1.0
No	8 (80%)	10 (100%)	
**Antibiotic consumption (current flock)**			
Yes	5 (50%)	5 (50%)	1.0
No	5 (50%)	5 (50%)	
**Reasons for antibiotic consumption**			
Treatment	10 (100%)	10 (100%)	0.775
Prophylaxis	1 (10%)	1 (10%)	
Metaphylaxis	2 (20%)	2 (20%)	
**Stock of antibiotics in farm**			
Yes	1 (10%)	1 (10%)	0.521
No	9 (90%)	9 (90%)	
**Use of antibiotics before their expiration**			
Yes	10 (100%)	10 (100%)	-
No	0 (0%)	0 (0%)	
**Are withdrawal periods applicable?**			
Yes	10 (100%)	10 (100%)	-
No	0 (0%)	0 (0%)	
**Antibiotic consumption after vet's prescription**			
Always	10 (100%)	10 (100%)	-
Usually	0 (0%)	0 (0%)	
Sometimes	0 (0%)	0 (0%)	
Rarely	0 (0%)	0 (0%)	
**Microbial culture test and antibiogram performed**			
Always	0 (0%)	0 (0%)	-
Usually	0 (0%)	0 (0%)	
Sometimes	10 (100%)	10 (100%)	
Never	0 (0%)	0 (0%)	
**Guidance by vet**			
Complete	10 (100%)	10 (100%)	-
Partial	0 (0%)	0 (0%)	
None	0 (0%)	0 (0%)	

Table S16. Characteristics of broiler farms participated in the study.

Farm	Season	Number of birds / flock	Age of flock	Type of breeding	Neighboring poultry farms	Type of disinfection	Temperature in barn (°C)	Ventilation / ammonia in barn	Stocking density (birds/m²)	Type of bedding	Consumption of food / water	Animal performances
CM1	Winter	57000	44	Intensive	No	CID 2000 / Virkon-s	20	Good / low	15	Rice hull	171000	Excellent
CM1	Summer	57000	38	Intensive	No	Kenosan / Virkon-s	20	Good / low	14	Sawdust	176700	Excellent
CM2	Winter	53000	46	Intensive	No	CID 2000 / ALDEKOL03	20	Good / low	14	Sawdust	159000	Good
CM2	Summer	56000	42	Intensive	No	Kenosan / Aldekol 03 FF	20	Good / low	14	Rice hull	179200	Good
CM3	Winter	29000	43	Intensive	Yes	Virocid	20	Not good / high	13	Rice hull	72500	Mediocre
CM3	Summer	20500	39	Intensive	Yes	Kenosan / Cid 20	20	Good / low	13	Rice hull	65600	Good
CM4	Winter	75000	45	Intensive	Yes	Virocid	22	Mediocre / mediocre	14	Rice hull	225000	Mediocre
CM4	Summer	66000	43	Intensive	Yes	Virkon-s / Virocid	20	Good / low	15	Sawdust	217800	Good
CM5	Winter	42000	45	Intensive	No	CID 2000 / Virkon-s / CID 20	20	Good / low	15	Rice hull	126000	Excellent
CM5	Summer	38000	41	Intensive	No	Kenosan / Virocid	20	Good / low	14	Straw	125400	Good
Ep.1	Spring	67320	45	Intensive	Yes	Virkon-s / Aldecol	31	Good / low	ND*	Straw	276210	Mediocre
Ep.1	Autumn	68340	47	Intensive	Yes	Virkon-s / Aldecol	31	Good / low	ND	Straw	279420	Good
Ep.2	Spring	24480	44	Intensive	Yes	Virkon-s / Aldecol	31	Good / low	ND	Rice hull	89700	Good
Ep.2	Autumn	24480	42	Intensive	Yes	Virkon-s / Aldecol	31	Good / low	ND	Rice hull	94620	Good
Ep.3	Spring	18360	43	Intensive	Yes	Virkon-s / Aldecol	31	Good / low	ND	Rice hull	71990	Good
Ep.3	Autumn	17544	42	Intensive	Yes	Virkon-s / Aldecol	31	Good / low	ND	Rice hull	66090	Excellent
Ep.4	Spring	16320	43	Intensive	Yes	Virkon-s / Aldecol	31	Good / low	ND	Rice hull	65050	Good
Ep.5	Spring	17340	45	Intensive	Yes	Virkon-s / Aldecol	31	Good / low	ND	Straw	75780	Mediocre
Ep.6	Autumn	15096	44	Intensive	No	Virkon-s / Aldecol	31	Good / low	ND	Straw	65480	Good
Ep.7	Autumn	8670	45	Intensive	No	Virkon-s / Aldecol	31	Good / low	ND	Straw	36820	Good

*ND = No data

Table S17. History of diseases in poultry flocks.

Farm	Season	History of diseases				
		Disease	Beginning (day)	Duration (days)	Therapy	Outcome
CM1	Winter	No	No	No	No	No
CM1	Summer	No	No	No	No	No
CM2	Winter	No	No	No	No	No
CM2	Summer	No	No	No	No	No
CM3	Winter	No	No	No	No	No
CM3	Summer	*E. coli*	4	4	Trimethoprim-sulfodiazine for 4 days	Therapy
CM4	Winter	No	No	No	No	No
CM4	Summer	*S. aureus*	21	4	Tetracycline for 4 days	Therapy
CM5	Winter	No	No	No	No	No
CM5	Summer	No	No	No	No	No
Ep.1	Spring	No	No	No	No	No
Ep.1	Autumn	No	No	No	No	No
Ep.2	Spring	No	No	No	No	No
Ep.2	Autumn	No	No	No	No	No
Ep.3	Spring	No	No	No	No	No
Ep.3	Autumn	No	No	No	No	No
Ep.4	Spring	No	No	No	No	No
Ep.5	Spring	No	No	No	No	No
Ep.6	Autumn	No	No	No	No	No
Ep.7	Autumn	No	No	No	No	No

Table S18. Antibiotic use in broiler farms.

Farm	Season	Antibiotics_last 3 months	Antibiotics_current flock	Reasons for antibiotic consumption	Are withdrawal periods applicable	Stock of antibiotics in farm	Use of antibiotics before their expiration	Antibiotic consumption after doctor's prescription	Microbial culture test and antibiogram performed	Guidance by vet
CM1	Winter	No	No	Therapy	Always	No	Yes	Always	Sometimes	Complete
CM1	Summer	No	No	Therapy	Always	No	Yes	Always	Sometimes	Complete
CM2	Winter	No	No	Therapy	Always	No	Yes	Always	Sometimes	Complete
CM2	Summer	No	No	Therapy	Always	No	Yes	Always	Sometimes	Complete
CM3	Winter	Doxycycline, Trimethoprim-Sulfodiazine	Trimethoprim-Sulfodiazine	Therapy, metaphylaxis	Always	No	Yes	Always	Sometimes (last one: *E. coli* O8, sensitive to antibiotics used)	Complete
CM3	Summer	No	Trimethoprim-Sulfodiazine	Therapy, metaphylaxis	Always	No	Yes	Always	Sometimes (last one: *E. coli* O78/K80, sensitive to antibiotics used)	Complete
CM4	Winter	Amoxicillin	Amoxicillin	Therapy, prophylaxis, metaphylaxis	Always	Yes (room temperature)	Yes	Always	Sometimes	Complete
CM4	Summer	No	Tetracycline	Therapy, prophylaxis, metaphylaxis	Always	Yes (room temperature)	Yes	Always	Sometimes (last one: S. aureus, sensitive to antibiotic used)	Complete
CM5	Winter	No	No	Therapy	Always	No	Yes	Always	Sometimes	Complete
CM5	Summer	No	No	Therapy	Always	No	Yes	Always	Sometimes	Complete
Ep.1	Spring	No	Amoxicillin	Therapy	Always	No	Yes	Always	Sometimes	Complete
Ep.1	Autumn	No	Sulfonamide	Therapy	Always	No	Yes	Always	Sometimes	Complete
Ep.2	Spring	No	No	Therapy	Always	No	Yes	Always	Sometimes	Complete
Ep.2	Autumn	No	No	Therapy	Always	No	Yes	Always	Sometimes	Complete
Ep.3	Spring	No	No	Therapy	Always	No	Yes	Always	Sometimes	Complete
Ep.3	Autumn	No	No	Therapy	Always	No	Yes	Always	Sometimes	Complete
Ep.4	Spring	No	Trimethoprim-Sulfodiazine	Therapy	Always	No	Yes	Always	Sometimes	Complete
Ep.5	Spring	No	Doxycycline	Therapy	Always	No	Yes	Always	Sometimes	Complete
Ep.6	Autumn	No	Doxycycline	Therapy	Always	No	Yes	Always	Sometimes	Complete
Ep.7	Autumn	No	Cephalexin	Therapy	Always	No	Yes	Always	Sometimes	Complete
